# Prospective Life Cycle Assessment Suggests Direct Reduced Iron Is the Most Sustainable Pathway to Net-Zero Steelmaking

**DOI:** 10.1021/acs.iecr.4c03321

**Published:** 2025-02-08

**Authors:** Arezoo Azimi, Mijndert van der Spek

**Affiliations:** Research Centre for Carbon Solutions, Heriot-Watt University, Edinburgh EH14 4AS, United Kingdom

## Abstract

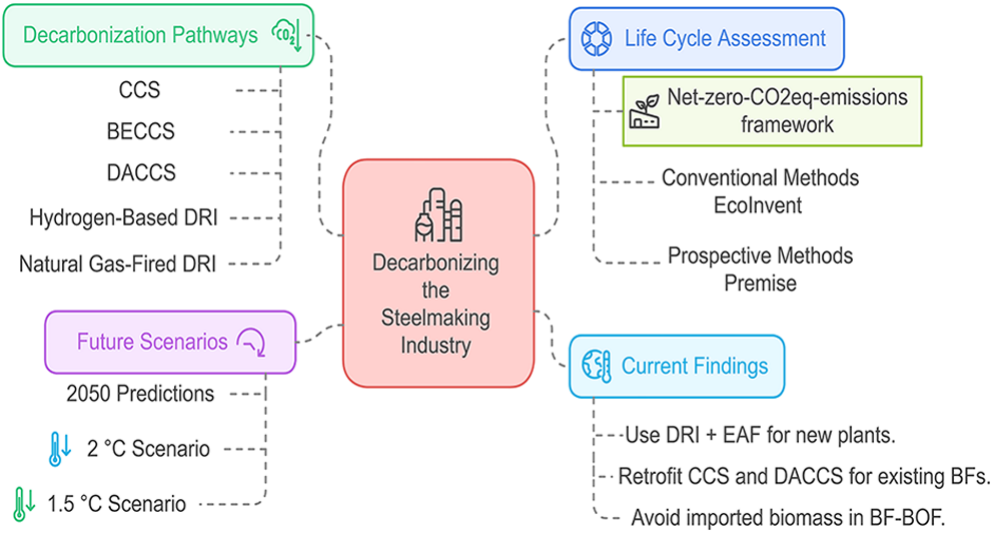

Decarbonizing the steel industry is essential due to its substantial contribution to climate change. This study explores pathways to achieve net-zero CO_2eq_ emissions in the iron and steelmaking industry while minimizing environmental burdens beyond climate change. We conducted a comprehensive attributional life cycle assessment using the net-zero-CO_2eq_-emissions framework, incorporating both conventional and prospective life cycle assessment methods, to evaluate various decarbonization strategies within the United Kingdom. All value chains were constrained to achieve net-zero CO_2eq_ emissions. Our findings indicate that, under a “current time” scenario, the natural gas-fired direct reduced iron with electric arc furnace is the most favorable option. This is mainly because hydrogen-based direct reduced iron production relies on the UK’s current electricity grid, which has a carbon intensity of 293.28 g CO_2eq_ per kWh. As greenhouse gas emissions decrease toward 2050 (approximately 70% for hydrogen-based direct reduced iron), the choice between natural gas and hydrogen will become increasingly region-specific. All net-zero-CO_2eq_ steelmaking case studies perform similarly on human health indicators, while the direct reduced iron with electric arc furnace options have 60–82% lower impacts on the ecosystem end point indicator than the blast furnace basic oxygen furnace routes.

## Introduction

The average per capita steel use worldwide has steadily increased from 150 kg in 2001 to around 220 kg in 2023. By 2050, steel use is projected to increase by approximately 20% compared to current levels, to meet the demands of a growing global population. The steel industry employs over 6 million people globally.^1^

Decarbonizing the steel industry is crucial due to its significant impact on climate change. The steel industry is highly energy- and emission-intensive, ranking first in CO_2_ emissions and second in energy consumption among industrial sectors. According to the World Steel Association,^2^ in 2021, the iron and steelmaking industry emitted 1.91 tonnes of CO_2_ per tonne of crude steel cast produced and consumed 20.99 GJ of energy per tonne. It is currently the largest industrial consumer of coal, which provides around 75% of its energy demand and is essential for producing coke, a key component in the chemical reduction of iron ore in blast furnaces.

To meet global climate goals, CO_2_ emissions from the iron and steel industry must decline toward net-zero by 2050. Strategies for achieving deep emissions reductions in steelmaking include CO_2_ capture and storage (CCS), fuel switching (e.g., hydrogen or bioenergy), direct electrification, and innovative process designs such as direct reduction of iron ore with hydrogen. Factors such as energy prices, technology costs, and the availability of raw materials will significantly influence the adoption of these technologies.^3^ For example, access to low-cost renewable electricity provides a competitive advantage to hydrogen-based steelmaking. Additionally, to achieve 2050 climate goals, the rapid deployment of technologies that are currently in early development stages is required. For example, the International Energy Agency (IEA) estimated that a new hydrogen-based direct reduction plant needs to be deployed every month once the technology is commercially available.^1^

The Western European steel industry is undergoing a significant transition from coal-based production methods to more sustainable power-based processes. This shift is being spearheaded by several countries committed to reducing carbon emissions. In the United Kingdom, a major transformation is underway, with the planned shutdown of two primary steelmaking sites operated by British Steel in Scunthorpe and Tata Steel in Port Talbot. These sites, which currently rely on traditional blast furnace and basic oxygen furnace (BF/BOF) processes, will be replaced by electric arc furnaces (EAFs) that focus on recycling scrap steel (while we note this will remove the UK’s primary steelmaking capability). This move represents a key step in reducing the carbon footprint of the UK’s steel industry. Germany is also making strides toward greener steel production. ArcelorMittal has announced plans to integrate hydrogen-based processes in its steelmaking plants in Hamburg, Bremen, and Eisenhüttenstadt by the mid-2020s. The latter two plants will transition to EAFs as part of this initiative. Additionally, ThyssenKrupp has committed to building a hydrogen-based direct reduced iron (DRI) plant in Duisburg by 2025, further solidifying Germany’s leadership in sustainable steelmaking.^4^ In France, the steel industry continues to prioritize emissions reduction through carbon capture, utilization, and storage (CCUS) technologies. The country operates five blast furnaces with a combined production capacity of approximately 15 million tonnes of primary steel annually. While France remains focused on CCUS,^4^ Spain is taking significant steps toward decarbonization with ArcelorMittal’s agreement to invest 1 billion euros in its BF/BOF steelmaking plant in Gijón. This investment will fund the development of a green hydrogen DRI unit, complemented by a hybrid EAF, marking a major shift in Spain’s steel production landscape.^5^ Sweden’s SSAB^6^ is leading a groundbreaking initiative called “Toward Fossil-Free Steel,” in collaboration with industrial and research partners. The project aims to transform Nordic strip production to achieve net-zero emissions by around 2030. As part of this effort, the SSAB plans to convert its Luleå and Raahe sites into mini mills with EAFs and rolling mills in Sweden and Finland, respectively. The company will further develop its Borlänge and Hämeenlinna plants to align with these new production processes. Belgium’s ArcelorMittal Gent^7^ is set to electrify and decarbonize its existing blast furnaces by utilizing captured CO_2_ emissions through a testing pilot carbon capture unit developed by Mitsubishi Heavy Industries (MHI). These emissions will be converted back into carbon monoxide (CO) using plasma technology from D-CRBN. This innovative process will reduce coal usage in blast furnaces and decrease the future need for green hydrogen. In The Netherlands, Tata Steel IJmuiden, the country’s only operating BF/BOF steel facility, is embarking on a significant transformation as part of the “Green Steel Plan.” By 2030, the site’s largest blast furnace, BF7, is planned to be replaced by an EAF, while a new DRI plant will take the place of one of the company’s coke-making facilities.^8^

Overall, the transition to green steelmaking is largely focused on two key strategies: retrofitting carbon capture, utilization, and storage (CCUS) technologies onto existing primary steelmaking plants (BF/BOF) or shifting to secondary steelmaking by using EAFs that recycle steel scrap. Another emerging approach involves combining DRI with EAFs to create a fully integrated, low-carbon steelmaking supply chain. Relying solely on EAFs for steel production presents challenges, particularly due to the dependency on scrap steel or imported sponge iron, which can lead to supply constraints. Moreover, the continuous recycling of steel can result in the accumulation of impurities, potentially compromising the quality of the final product over multiple cycles.

Existing research indicates that retrofitting CCUS to the traditional BF/BOF steelmaking process has a decarbonization potential of up to 45%.^9^ On the other hand, integrating bioenergy with carbon capture and storage (BECCS) into the BF/BOF route reduces emissions to only 0.1 t of CO_2_ per tonne of hot rolled coil (HRC). In contrast, the DRI/EAF steelmaking route has been shown to achieve net negative life cycle CO_2_ emissions by integrating BECCS, at negative 0.5 tonnes of CO_2_ per tonne of HRC.^10^

Despite these advancing insights in decarbonization potential, the literature still lacks a comprehensive study on how to reach net-zero CO_2eq_ emissions in iron and steelmaking in the most environmentally benign manner, i.e., taking stock of the environmental indicators beyond greenhouse gases. Moreover, there is limited exploration of the potential environmental trade-offs associated with different decarbonization strategies, critically hindering decision makers to select the steel decarbonization options that are most sustainable, or that suit their environmental targets best. This study addresses these gaps by conducting a full attributional life cycle assessment (LCA) on net-zero-CO_2eq_-emissions of iron and steelmaking for the United Kingdom. This was done by integrating the net-zero-CO_2eq_-emissions framework postulated by Mazzotti and co-workers^11^ and further developed for LCA by van der Spek and co-workers,^12,13^ with prospective LCA for selected (full) decarbonization configurations. The results can aid decision makers in selecting decarbonization strategies suiting their needs and targets, and robust against future changes in the wider industrial system iron and steelmaking operates in.

## Process Descriptions and Interventions

**Figure 1:**
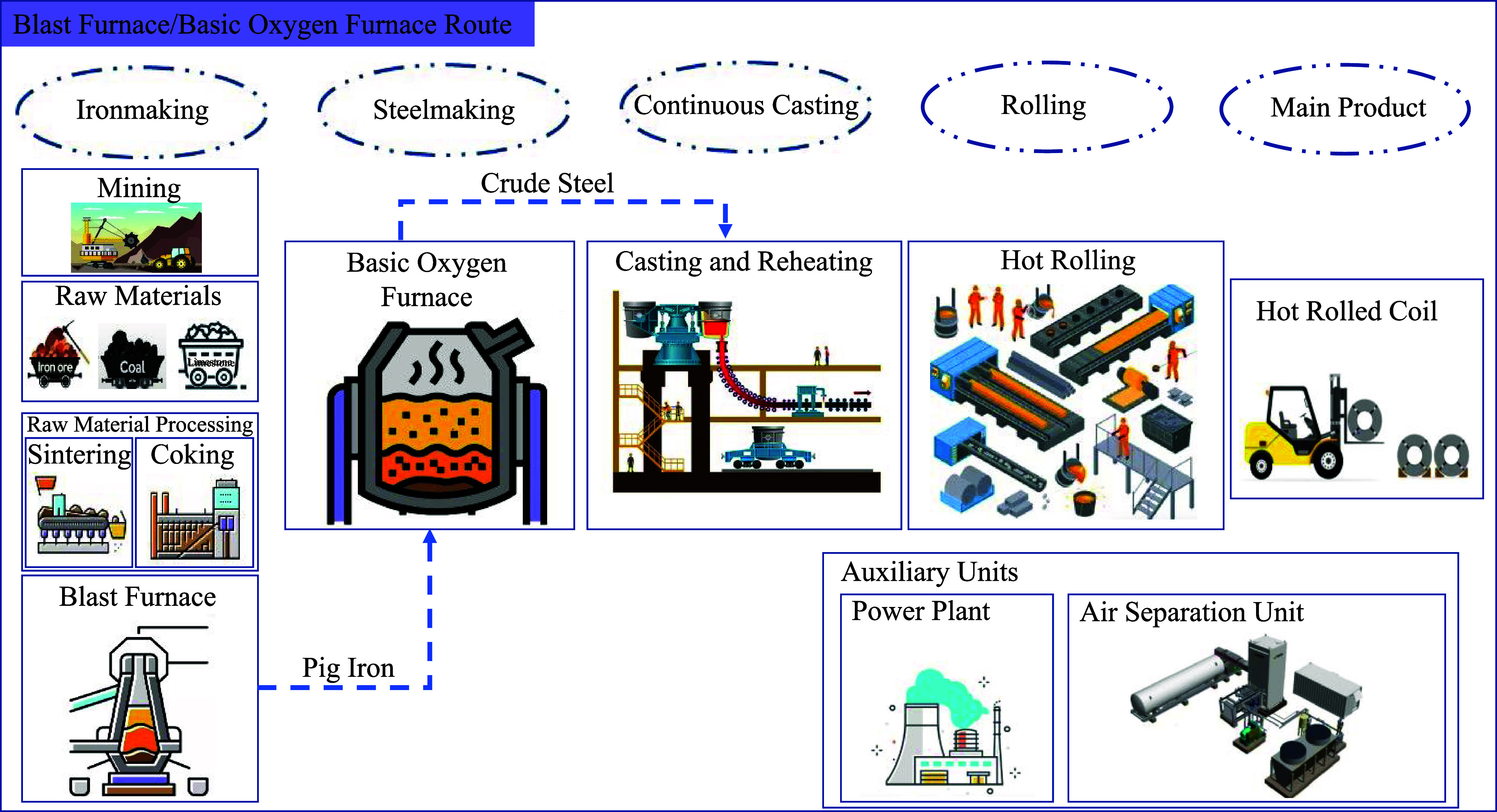
Schematic diagram of hot rolled steel production via the blast furnace/basic oxygen furnace route, encompassing all processes from mining and ironmaking to hot rolling.

**Figure 2:**
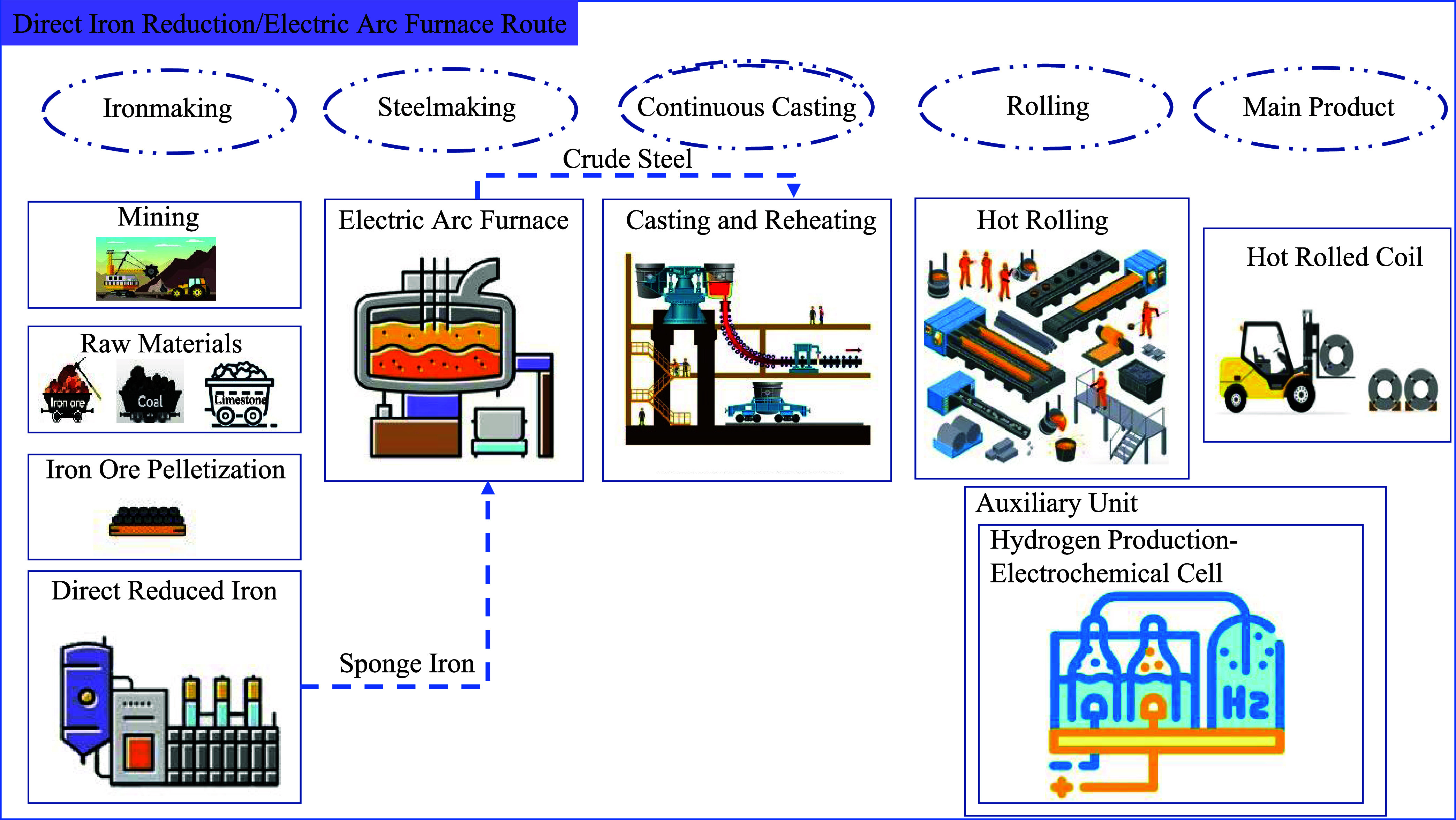
Schematic diagram of hot rolled coil production via the direct reduced iron/electric arc furnace route, encompassing all processes from mining and ironmaking to hot rolling.

Crude steel production follows roughly four steps: raw material acquisition, raw material preparation, ironmaking, and finally steelmaking. These stages are followed by continuous casting and rolling to produce various steel products. The two main routes for iron and steelmaking are the traditional blast furnace/basic oxygen furnace route, known as the primary route, and the more recent direct reduced iron/electric arc furnace route. These two routes are investigated here, with a focus on the primary and secondary steelmaking processes, as electric arc furnaces do not produce primary iron, such as pig iron or sponge iron. A significant challenge of relying solely on electric arc furnaces is the limited and inconsistent supply of high-quality scrap, which can introduce impurities and compromise the consistency of the steel properties. Consequently, electric arc furnaces may need to partially rely on imported sponge iron as a feedstock. These routes differ mainly in the ironmaking and steelmaking stages. Figures 1 and 2 provide detailed descriptions of both.

### Blast Furnace/Basic Oxygen Furnace Description, Steps, and Processes

The blast furnace–basic oxygen furnace route is currently responsible for 71.1% of global steel production.^2^ It requires a significant number of raw materials, including coal, limestone, and iron ore. Iron ore will be deployed in different forms such as iron ore fines, iron ore pellets, and lump iron ore based on the requirement of each process. Lump ores are directly utilized in the blast furnace, while a portion of the iron ore is processed through pelletizing to produce pellets for the blast furnace feed. Coal is transformed into coke in coke ovens, and limestone is converted to lime in lime kilns. The processed materials are then combined and sent to a blast furnace for primary ironmaking.

Ironmaking stage occurs in a BF which operates at very high temperatures, ranging from 1500 to 2300 °C. Iron ore pellets and sinter are layered with coke and limestone and sometimes lime. Cokes function both as a fuel and as a reducing agent. The intense heat and chemical reactions in the furnace reduce iron ore to molten iron (or pig iron) and produce carbon dioxide and slag. The pig iron is tapped from the furnace’s bottom, while the slag, formed from the fluxes combined with impurities, floats on top of the molten iron and is removed for waste treatment. The blast furnace gas (BFG) generated during the process consisting of CO_2_ (21.86 vol %) and CO (22.10 vol %) is utilized as a fuel for electricity generation in most steel plants that have on-site power plants.

In the subsequent steelmaking stage, a basic oxygen furnace is used to convert molten iron from the blast furnace into steel. The BOF operates by blowing high-purity oxygen, produced on-site in an air separation unit, onto molten iron. This oxygen reacts with carbon, silicon, and manganese in the pig iron to produce carbon dioxide and other gases. The temperature in the BOF is carefully controlled, typically between 1600 and 1700 °C, to ensure the steel achieves the desired chemical composition. The produced slag is removed separately and sent to waste management for different applications, such as road construction. Additionally, Basic Oxygen Furnace Gas (BOFG) is used as fuel for electricity generation within the on-site power plant. Finally, the molten steel is transferred to continuous casting for solidification and hot rolling for shaping into the finished products.

### Direct reduced iron/Electric arc furnace Description, Steps and Processes

This route is gaining popularity due to its flexibility and potential for integrating renewable energy sources, potentially leading to a lower environmental impact. Iron ore fines must be pelletized to ensure consistent quality prior to the reduction process. In this route, iron ore reduction can be achieved using coal, natural gas, or hydrogen in the DRI shaft. The reducing gas is introduced at high temperatures (around 800–1050 °C), removing the oxygen from iron ore pellets, and producing solid sponge iron known as DRI. The DRI is then charged into the EAF where it is heated between 1500 and 1700 °C^14,15^ which melts the DRI and produces molten steel. The energy for this process comes from the grid or renewable sources such as solar or wind power, if available. A significant portion of the required energy is provided by injection of oxygen and coal. This method also allows for the efficient recycling of scrap steel directly in the EAF, further reducing the demand for virgin raw materials. Additionally, the modular nature of, especially, EAF plants makes them well suited for localized production, reducing transportation emissions and costs.

### Interventions

To compare the performance of these two iron and steelmaking routes under a net-zero constraint, different interventions were designed and combined with each route to determine which combination shows optimal environmental performance and may achieve net-zero-CO_2eq_-emissions. Given that few single interventions will reduce net CO_2eq_ emissions to zero, we included combinations of CO_2_ reduction and CO_2_ removal technologies, e.g., partial coal replacement with biofuel combined with CCS, or end-of-pipe CCS combined with direct air CO_2_ capture and storage (DACCS). In total, 12 different case studies were created: seven focused on the BF/BOF route and the remaining five on the DRI/EAF route. These case studies are listed in Table 1. Detailed descriptions of the interventions are provided.

**Table 1:** Overview of the Case Studies Investigated

steelmaking technology	case studies
BF/BOF	BF/BOF (base case)
	BF/BOF (CCS)
	BF/BOF (biomass)
	BF/BOF (BECCS)
	BF/BOF (CCS + DACCS)
	BF/BOF (biomass + DACCS)
	BF/BOF (BECCS + DACCS)
DRI/EAF	DRI-NG/EAF
	DRI-H_2_/EAF
	DRI-NG/EAF (CCS)
	DRI-NG/EAF (CCS + DACCS)
	DRI-H_2_/EAF (DACCS)

#### CO_2_ Capture and Storage (CCS)

A solvent-based postcombustion capture plant with 90% efficiency using Monoethanolamine (MEA) was assumed here, for which the data inventory was sourced from the 2013 IEAGHG report on decarbonizing steel production.^16^ After capture, the CO_2_ was assumed to be sent for compression, transport, and geological storage (using inventories from Qiu et al.^17^). This capture plant is designed to capture emissions from coke oven batteries, blast furnace hot stoves, lime production plant, and steam generation plant in the BF/BOF route and from the direct reduction shaft in the DRI/EAF route. When retrofitted on the steel mill, the overall electricity and fuel consumption of the plant will increase to drive the capture and compression plants. For BF/BOF (Base case), electricity is generated in a power plant that utilizes flue gases from coke ovens (COG), BFG, and BOFG to produce steam (see Figure S1). However, in the BF/BOF (CCS) case study, the internal gas supply will be insufficient, as it will be redirected to the on-site steam generation plant to meet the steam requirements of the capture process (see Figure S1). Consequently, the power plant will rely on natural gas to generate electricity. On the other hand, in the case of DRI-NG/EAF (CCS), the CCS plant already takes the energy from natural gas, as there is no internal combustion gas stream available. The capture plant in this case only captures the emissions of the DRI process.

The CCS plant does not only capture 90% of CO_2_ emissions but is assumed to also remove 90% of SO_*x*_ emissions, 1.25% of NO_*x*_ emissions, and 50% of particulate matter from each process it is capturing from.^18^

#### Fuel Substitution with Biomass

The biomass cases assume that wood chips are imported from Quebec, Canada to the United Kingdom, given the lack of sufficient biomass supply options from the UK itself. The transportation and land use change and transformation of biomass into chips are included in our modeling. Upon arrival, the wood chips undergo torrefaction at the steel mill site. Torrefaction occurs in an inert or oxygen-deficit environment at temperatures between 200 and 300 °C, following.^19^ During this process, the moisture content of biomass decreases and some of the hydrogen- and oxygen-containing organic components of organic compounds are thermally decomposed, releasing volatile organic compounds. The final product of the torrefaction process is a solid, uniform product with lower moisture and approximately 30% higher energy content per unit of mass than the original wood chips.

The torrefied wood pellets were substituted at different rates in each case study, as reported in Table 2. Some fossil-based materials serve as energy sources, while others act as reducing agents, making their carbon content particularly important. In the BF/BOF (Biomass) case study, the coking coal and coke were substituted with torrefied wood pellets based on their respective carbon content ratio, and the Pulverized Coal Injection (PCI) coal was substituted based on the lower heating values (LHV) of the materials. The carbon content and LHV of each material are reported in Table 3.

**Table 2:** Biomass Substitution Rates for the Biomass and BECCS Cases

case study	bioenergy use
BF/BOF (biomass)	5% of coking coal in coke ovens, 50% of coke in the sinter plants, 100% of the PCI coal in blast furnace
BF/BOF (BECCS)	as in the biomass case, plus 100% of natural gas in the steam generation plant

**Table 3:** Energy and Carbon Contents of Fuels Used in This Study

fuel type	LHV (MJ/kg)	carbon content (wt %)	reference
coal	28.2	87	(16)
coke		88.05	(16)
natural gas	48		EcoInvent 3.9.1
torrefied wood pellets	18.64	72.07	(19)

#### Bioenergy with CCS (BECCS)

BECCS is a process that integrates substituting fossil-based materials with biofuels, here torrefied wood pellets, and then captures and safely stores the resulting CO_2_ emissions. In this case, the power plant needs to be larger than that in the BF/BOF (Biomass) case study. Additionally, natural gas used in the steam generation plant is replaced with biomass, based on the LHV ratio of the respective fuels, as detailed in Table 3. The CCS plant will capture 90% of both fossil CO_2_ emissions from each plant and 90% of biogenic emissions based on the carbon content of the materials that have been substituted. These captured biogenic emissions will have negative environmental burdens in the LCA. Capture rates of SO_*x*_, NO_*x*_, and particulate matter are assumed to be the same as in the CCS cases.

#### Hydrogen Production via Electrolysis

Here, hydrogen produced through the electrolysis of water in an electrolyzer is used in the DRI shaft in place of natural gas or coal for reducing iron ore. The production of hydrogen via electrolysis requires substantial amounts of freshwater and electricity. If this electricity is sourced from the grid and is not derived from renewable or low-carbon sources, the environmental impact can be significant, while renewably sourced electricity can result in improved environmental impacts.

#### Direct Air CO_2_ Capture and Storage (DACCS)

Here, an absorbent-based DACCS with potassium hydroxide (KOH) was selected to offset any remaining emissions that the other interventions could not remove. The primary energy source in this DACCS plant is natural gas, supplemented with grid electricity for pumps, fans, and CO_2_ compression. We selected NG-fired DACCS since steel plants often have natural gas on their sites already. The inventory data for this process were sourced from Qiu et al.,^17^ where the reader can also find more information on the DACCS process considered. Details of the comparison are provided in Figure S5.

## Methodology

### Net-Zero-CO_2eq_-Emission Framework

To fulfill the purpose of this study, the net-zero-CO_2eq_-emission framework for life cycle analysis (LCA) was utilized, previously postulated in refs (12,13) which was here complemented with prospective LCA (pLCA) (Prospective LCA (pLCA) section). The key objective of the net-zero framework for LCA is to analyze the environmental performance of different, full, decarbonization pathways, on a like-for-like basis, i.e., constraint by net-zero CO_2eq_ emissions. It involves measuring and identifying greenhouse gas emissions across all stages of products or processes’ life cycle—from raw material extraction through production, use, and end-of-life treatments—and then reducing these emissions to net-zero by employing available decarbonization options (e.g., energy efficiency measures, electrification, fuel switching, or CCUS and CDR technologies). Following this, the study investigates the remaining (non-climate change) environmental trade-offs associated with the different decarbonization pathways and seeks to find those interventions that provide the complete CO_2eq_ emissions abatement while minimizing other negative environmental impacts. By refining these processes, the framework aids in designing the most efficient and sustainable interventions, ensuring both the lowest CO_2eq_ emissions and the most favorable overall environmental outcomes.^13^

This framework follows the LCA guidelines^20^ with some improvement in each step. It starts with goal and scope definition and ends with comparing the midpoint or end point impact indicators.

#### Define Goal and Scope of the System

The goal of the study, system boundaries, and functional unit must be defined to align with the objective of net-zero CO_2eq_ emissions throughout the life cycle of the incumbent system. The functional unit should reflect the primary product of the incumbent system, typical for the nondecarbonized production system. Finally, like in any LCA, all emissions and discharges to air and water, the use of fuels and mineral resources for construction and transportation, and other environmental aspects need to be included and linked to relevant impact categories, ensuring a comprehensive environmental analysis.

#### Build Life Cycle Inventory (LCI)

Data on energy use, raw material consumption, emissions to air, water, and soil, and other environmental aspects should be included in the system boundaries. Foreground data, including the mass and energy balance, should be obtained for each specific process. Background data, such as raw material extraction, transportation, etc., can be sourced from available databases like EcoInvent.

#### Identify CO_2eq_ Emissions in the Base Production System (CO_2_ Positive)

Once data on the main incumbent system is collected, the total value chain within the system boundaries should be thoroughly scrutinized to identify the CO_2eq_ emissions of the nondecarbonized system. Identifying the sources of these emissions is crucial for designing effective mitigation options because the mitigation strategies will be designed in part 4 based on the type and volume of the emissions.

#### Introduce Measures/Technologies to Return CO_2eq_ Emissions to the System

Decarbonizing options need to be introduced to reduce the emissions identified in step 3. The main mitigating measures implemented to the system may include CCUS and CCS options, but there will be remaining GHG emissions throughout the system. The decarbonizing technology for any remaining emission needs to be selected based on the scope of study and can include any decarbonization measure, e.g., electrification, fuel switching, and carbon dioxide removal (CDR) technologies. CDR options can be implemented to offset any remaining or dispersed or otherwise hard-to-abate emissions. Inventory data for each decarbonization option are collected and added to the LCI. The resulting system will have net-zero CO_2eq_ emissions.

#### Calculate Performance Indicators, e.g., LC Midpoint Indicators for Current Time

Once the GHG emissions of all case studies are reduced to net-zero, evaluating impact categories other than climate change using typical life cycle assessment methods becomes necessary. A key thing to note is that, like in any LCA study, the environmental impact categories to study are selected based on the types of emissions and details included in the inventory. For example, if the LCI provides data on sulfur oxide emissions, it is meaningful to include acidification indicators, while this may be less meaningful if data on sulfur emissions is unavailable.

#### Calculate Performance Indicators for Future Scenarios

One of the criticisms of the net-zero framework was that it evaluates how a foreground system needs to be designed for the total value chain (foreground and background) to reach net-zero while using a background life cycle inventory database that represents the current, non-net-zero, situation. This implies that additional decarbonization measures are implemented to mitigate emissions from the background system, while this may be unnecessary in a future, net-zero economy. The recent development of the open access software Premise^21^ allows to solve this issue: Premise adjusts existing LCI databases to represent different future states, for different points in time, following scenarios that combine the so-called shared socioeconomic pathways (SSPs^22^) and representative concentration pathways (RCPs^23^), as modeled by integrated assessment models (the type of models calculating the IPCC scenarios). It uses the projections of these integrated assessment models to predict what the environmental footprint of selected activities will be for a selected scenario and given points in time. This means we can apply the net-zero LCA framework for scenarios that aim to reach (approximately) net-zero CO_2eq_ emissions by midcentury, e.g., combinations of the SSP2 with RCPs 1.9 or 2.6. The Results and Discussion section will illustrate how the use of future-adjusted background databases will change the environmental impact and trade-offs of different pathways to net-zero steelmaking, compared to using “current” databases (here, EcoInvent v3.9.1).

#### Evaluate the Systems Using the Calculated Performance Indicators

The final step is to conduct a comparative assessment of the designed interventions focusing on non-climate change environmental impacts, given that the systems are constrained to net-zero CO_2eq_ emissions. This assessment is applied to both current and future scenarios and needs to consider priority environmental concerns to the practitioner or study audience (e.g., different countries may have different environmental priority areas), allowing selection of the system whose environmental footprint best matches a desired situation.

### Goal and Scope, System Boundaries

**Figure 3:**
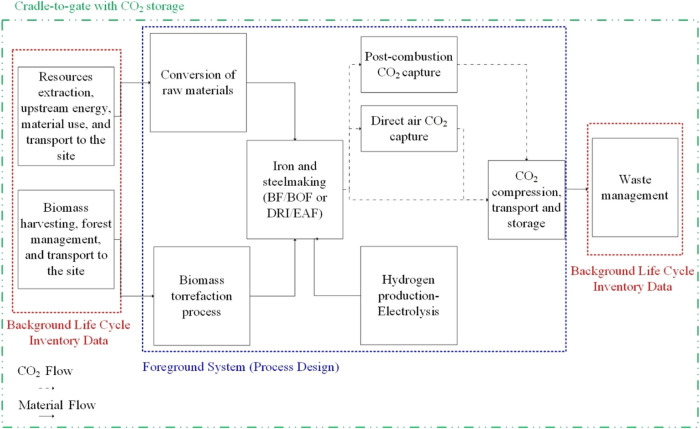
Cradle-to-gate with CO_2_ storage LCA system boundaries for the net-zero steelmaking cases.

In this study, an attributional LCA was conducted to evaluate the potential environmental impacts of the integrated system. The system boundary for the net-zero-CO_2eq_ steelmaking cases is outlined in Figure 3. The cradle-to-gate with the CO_2_ storage boundary includes upstream processes such as raw material extraction, transportation to the site, and their transformation to feedstock ready for ironmaking (either BF or DRI) and subsequent steelmaking (BOF or EAF). It also includes downstream processes like casting, reheating, and hot rolling. The BF/BOF route includes electricity production and an air separation unit, while the DRI/EAF route includes an electrolytic hydrogen production unit. To ensure comprehensiveness, the main product selected is Hot Rolled Coil (HRC), and the functional unit is 1 tonne of HRC produced. It is assumed that the steel mill is in the United Kingdom. Full (process and infrastructure) attributional LCA was conducted to study the performance of each case study. To achieve this, foreground inventories were developed for the respective case studies, based on their process designs and sourcing material and energy flows from the open literature (see LCI file in the Supporting Information). The open-source package Brightway with the Activity Browser GUI were used to unify the approach and LCIA methods across the current and prospective LCAs.

### Treatment of Biogenic Carbon Emissions

The treatment of biogenic carbon emissions and removals was approached as follows: CO_2_ uptake during biomass growth is accounted for by a negative biogenic carbon flow. Upon biomass combustion, the released biogenic carbon is included in the system’s overall CO_2_ emissions, rendering the process carbon-neutral. However, if biogenic carbon is captured and permanently stored in geological sites, as in the case of BECCS, then it is considered carbon-negative. Consequently, if the amount of atmospheric carbon removed and stored exceeds the GHG emissions generated throughout the life cycle of the process, the overall system can achieve a carbon-negative status.

### Life Cycle Inventory (LCI)

Foreground LCI data was collected from different literature sources indicated in Table 4. The detailed mass and energy balance for all case studies are provided in the LCI data file in the Supporting Information, while a summary of energy and resource demands for the ironmaking and steelmaking processes is given in Table 4. For the “current” situation, background data including data on energy production, raw material extraction, various types of transportation, and waste management was taken from EcoInvent v.3.9.1, system model “allocation, cutoff by classification”.

**Table 4:** Summary of Input Parameters for the Ironmaking Processes Studied (Per Tonne of HRC)

parameter	blast furnace	basic oxygen furnace	DRI-NG	DRI-H_2_	electric arc furnace	steel finishing and rolling
iron ore requirement	487 kg iron ore, 1110 kg sinter	5.50 kg iron ore, 974 kg pig iron^a^	1738.80 kg as iron pellets	1502.28 kg as iron pellets	64.80 kg iron scrap, 1090.8 kg sponge iron	none
fossil fuel demand	367 kg coke, 164 kg hard coal	none	306.55 m^3^ natural gas	44.06 m^3^ natural gas	2.94 m^3^ natural gas, 10.15 kg hard coal	none
flux demand	13.30 kg limestone	11 kg dolomite, 70 kg lime	none	none	27 kg quicklime	5.50 kg quicklime
oxygen demand	68 kg	79 kg	none	none	14.58 kg (inputted as electricity consumption for oxygen production 41.79 kWh/t HRC)	none
water demand	0.87 m^3^	0.42 m^3^	65.88 m^3^	683.42 m^3^ (including H_2_ production)	1.06 m^3^	2.914 m^3^
electricity demand	102.90 kWh (generated in an onsite power plant)	21.60 kWh (generated in an onsite power plant)	146.34 kWh	3780 kWh (including H_2_ production)	818.64 kWh	143.70 kWh
main data source	(16)	(16)	(24)	(24)	(24)	(16)

a Steel scrap has been used in the BOF, and the amount of pig iron is adjusted accordingly.

### Prospective LCA (pLCA)

In pLCA, the goal and scope, as well as the foreground data in the LCI, remain consistent with those used in the current time LCA. However, the background data vary according to the future scenario (SSP/RCP combination) used. Sacchi et al.^21^ introduced the Premise software to generate prospective inventory databases for pLCA adjusting activities in current databases like EcoInvent to modeled IAM scenarios. IAM scenarios are detailed narratives and quantitative models that describe possible futures based on different assumptions about policies, technologies, societal changes, and economic development.

In this study, for prospective LCA, two scenarios from the IAM model REMIND were used: SSP2 (Middle of the Road)-RCP2.6 and SSP2-RCP1.9. These scenarios help analyze the potential outcomes and impacts of middle-range socioeconomic developments combined with ambitious climate policies aimed at significant reductions in GHG emissions and reach net-zero CO_2eq_ emissions roughly by 2050, commensurate with EU and UK policy targets (and thus of relevance to this study). The background LCI databases for these scenarios were generated using Premise v1.8.2. dev3 using the ScenarioLink plugin in Activity Browser. In Premise, SSP2-PkBudg1150 is equivalent to the REMIND SSP2-RCP2.6 pathway, while SSP2-PkBudg500 is equivalent to REMIND SSP2-RCP1.9 pathway.

### Environmental Impact Categories and LCIA Methods

Studying the environmental trade-offs of the system is essential to avoid misjudging the designed interventions. The LCI developed here includes mass and energy flows, as well as the embodied carbon of infrastructures, SO_*x*_, NO_*x*_, and particulate matter (PM) emissions from the processes, slag and sludge formation and treatment, biomass harvesting, and land transformation. This allowed us to quantify the impact categories Land Use (LU), Material Resources Scarcity (MRS), Water Consumption (WC), Ozone Depletion (OD), Photochemical Oxidant Formation (POF), Particulate Matter Formation (PMF), Terrestrial Acidification (TA), Freshwater Eutrophication (FE), and Marine Eutrophication (ME), in addition to Climate Change. Climate change impacts were calculated using IPCC 2021, while the other impact categories were assessed using ReCiPe Midpoint (H) v1.03.

## Results and Discussion

### Non-Net-Zero Case Studies

**Figure 4:**
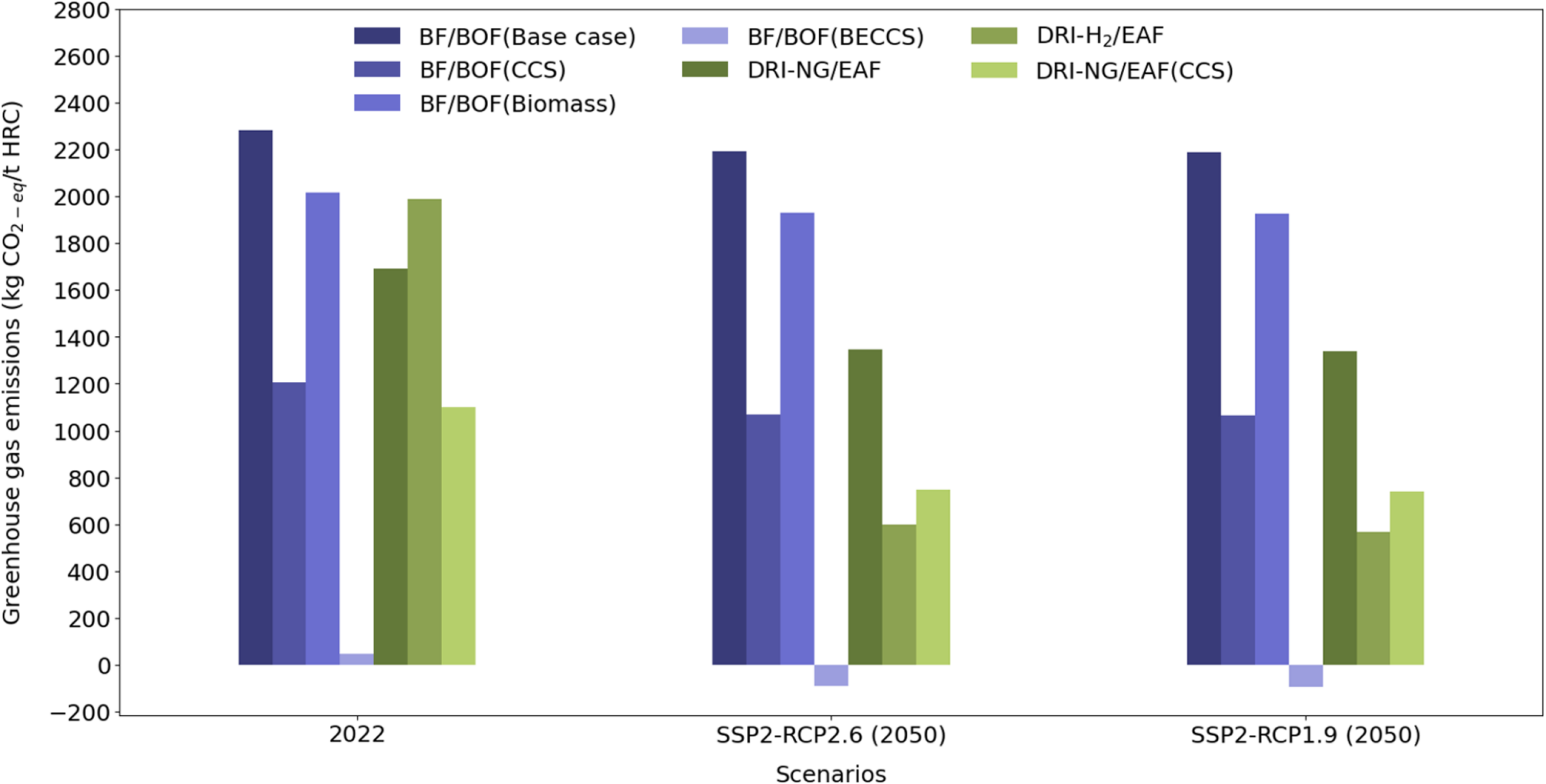
In a current (2022) scenario, in non-net-zero case studies neither fuel substitution with biomass nor BECSS renders full decarbonization in the BF/BOF route, and the DRI-H_2_ case study emits more than natural gas-fired DRI due to the high electricity consumption which comes from a nondecarbonized grid. However, the BF/BOF (BECCS) case study reaches net negative in future scenarios.

The conventional LCA results (Figure 4, first cluster on the left) confirm that neither the four BF/BOF case studies nor the three DRI/EAF cases achieve net-zero CO_2eq_ emissions under the current (2022) conditions. The base BF/BOF process emits approximately 2281 kg CO_2eq_ per tonne of HRC. When postcombustion CCS technology is retrofitted to the base case, emissions are reduced by nearly half (while Figure S10 shows that, when also the power plant emissions are captured and stored, the CO_2eq_ emissions are reduced to approximately 1000 kg CO_2eq_/t HRC). Looking at the third bar in the left cluster of Figure 4, it is evident that substituting fuels and fluxes with biomass does not significantly lower the emissions. This is largely due to the small amount of coal and cokes that can be replaced with biomass (while still rendering the process operable and the outputs of sufficient quality) and the inclusion of upstream emissions associated with biomass harvesting, transporting, and conversion within the system boundary of this LCA. The fourth bar in the left cluster shows that the BECCS case study reduces the emissions of the BF/BOF route to nearly net-zero (about 50 kg of CO_2eq_ per tonne of HRC) in the current time.

Turning to the DRI/EAF case studies (the last three bars of the first cluster on the left in Figure 4), it is observed that the emissions from the DRI-NG/EAF case study are lower than those from the H_2_-DRI/EAF case in a 2022 system. The total life cycle CO_2eq_ emissions for non-net-zero DRI-NG/EAF and DRI-H_2_/EAF are 1691 and 1989 kg per tonne of HRC, respectively. This discrepancy arises from the high energy consumption required for hydrogen production, using a current UK electricity mix with a CO_2_ intensity of 293.28 g of CO_2eq_ per kWh (based on EcoInvent 3.9.1). Thus, if the electricity for electrolysis is not sourced renewably, the greenhouse gas emissions from hydrogen-based DRI can exceed those from natural gas-based DRI. The retrofitting of CCS to the DRI-NG/EAF reduced the emissions only by one-third in the 2022 scenario.

The 2050 scenarios, represented by the middle and right clusters in Figure 4, show similar emission levels across all case studies compared to the current scenario, except for DRI-H_2_/EAF. In the SSP2-RCP1.9 scenario, emissions are slightly lower than in the SSP2-RCP2.6 scenario as expected, but the difference is negligible. This is chiefly because the foreground data for all case studies were kept static across both conventional and prospective LCAs, and the minor differences between the two 2050 scenarios stem from changes in the background data specific to each scenario.

In both prospective scenarios, the BF/BOF (BECCS) case study surpassed net-zero GHG emissions and became slightly net negative as in Figure 4, and therefore, this intervention will not need any further carbon offsets. The emissions of the other three BF/BOF case studies are almost the same as for the 2022 scenario.

On the other hand, the overall emissions of the DRI-H_2_/EAF case study drop significantly below those of the DRI-NG/EAF case study, compared to the conventional LCA results. In the prospective scenarios, the overall emissions of the DRI-H_2_ process fall from around 1989 (current scenario) to about 599 kg of CO_2eq_ per tonne of HRC. This is lower than the emissions of DRI-NG/EAF case study which emits around 1347 kg of CO_2eq_ per tonne of HRC in future scenarios. This reduction is attributed to the assumption that hydrogen is produced using the projected 2050 electricity mix in the UK. The carbon intensity of the UK grid in this scenario in 2050 will be around 15.7 g of CO_2eq_ per kWh (as per Premise).

### Net-Zero Case Studies-Hotspot Analysis

Above, we showed how neither CCS nor biomass substitution alone can achieve full decarbonization in both conventional and prospective scenarios except for BECCS (and only in the 2050 scenarios). To address any remaining emissions and ensure net-zero CO_2eq_ emissions, DACCS was added to each case study. The total GHG emissions of each case study were broken down to each subprocess to allow a better understanding of carbon emission hotspots.

#### Conventional LCA

**Figure 5:**
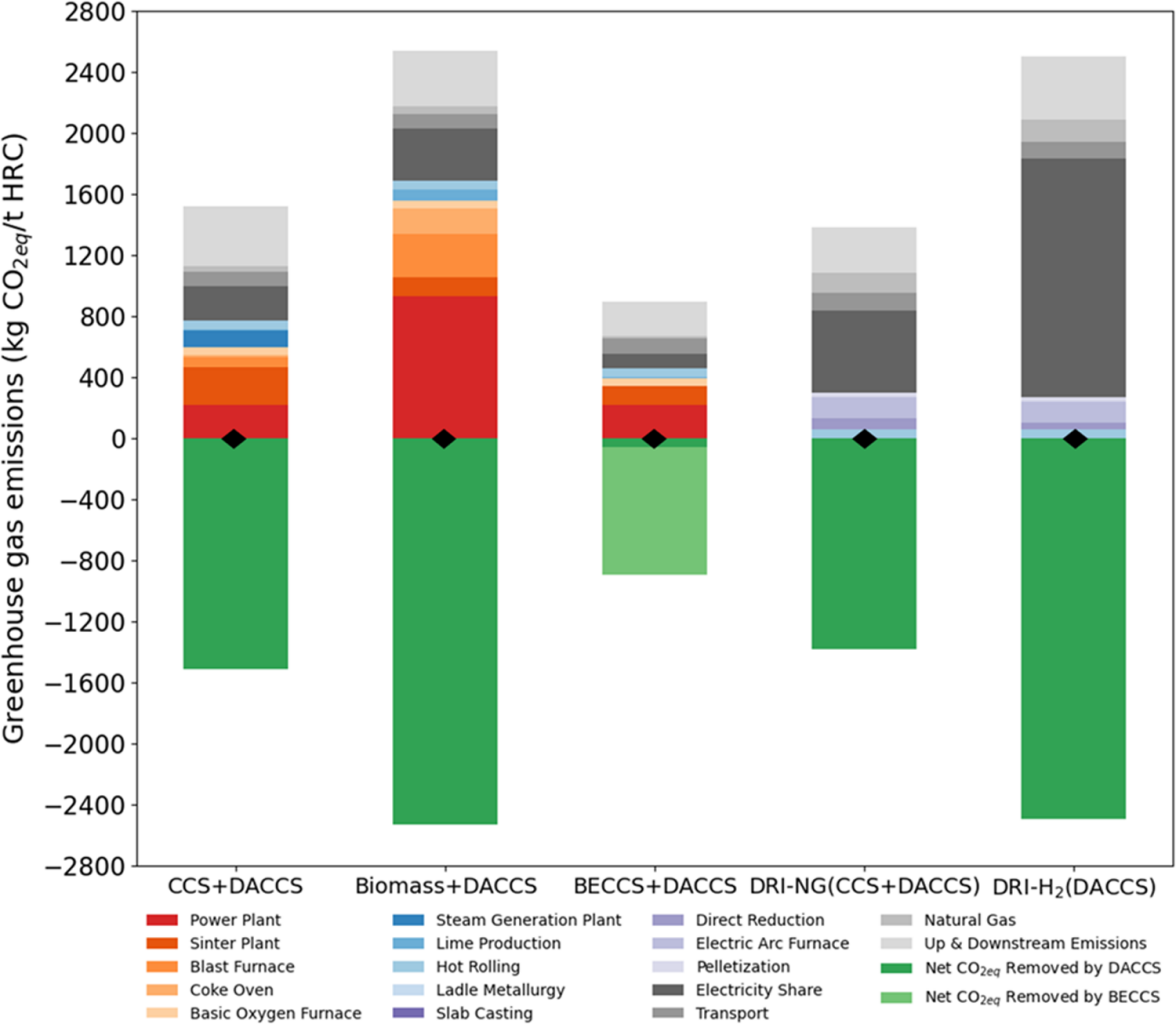
2022 scenario GHG emissions (kg of CO_2eq_/tonne of HRC) for the net-zero case studies broken down into the origin of the emissions. Note that, in the current time, the BF/BOF (BECCS) case study is almost net-zero but still needs small amounts of DACCS as offset. (⧫) shows the net-zero CO_2eq_ emissions points.

Figure 5 shows the CO_2eq_ breakdown for the net-zero steelmaking cases in a 2022 scenario. Note that the overall CO_2eq_ emissions that require offset increase across the case studies, compared to the non-net-zero cases discussed in the previous section. This is due to the addition of DACCS to offset residual emissions, which necessitates additional heat and electricity and contributes infrastructure emissions, collectively altering the emission baselines.

In the BF/BOF case studies (represented by the first three stacked bars on the left), the primary source of emissions is the power plant (220 kg of CO_2eq_ per tonne of HRC). In the BF/BOF (CCS + DACCS) scenario, CCS technology captures emissions from the coke oven batteries, blast furnace hot stoves, steam generation plant, and lime kilns, significantly reducing emissions from these sources. However, the power plant in the CCS scenarios is larger than in the cases without capture plant because it must supply additional electricity for the capture process. The energy for electricity production on the site in the BF/BOF (Base case) comes from internal gases such as COG, BFG, and BOFG, however, in the BF/BOF (CCS) case study all of these internal gases are redirected to the steam generation plant to produce the required steam for the capture plant, and the power plant will be generating electricity using natural gas (see the Figures S1 and S2). The share of GHG emissions for the power plant in the non-net-zero BF/BOF (base case) is around 926 kg per tonne of HRC. This share decreases in the cases with CCS, i.e., BF/BOF (CCS) and (BECCS) case study, even though the size of power plant is increased. This anomaly is because the power plant in the cases with CCS is now fed with natural gas and the LHV of natural gas is higher than internal gases, so we need less of that, then it emits less CO_2_.

In the BF/BOF (Biomass + DACCS) scenario (the second stacked bar on the left), overall emissions are higher than in the other cases. This is partially due to the higher power plant emissions and partially because of the emissions associated with biomass harvesting, transportation from Canada and Quebec to the UK, and conversion to torrefied wood pellets. Nevertheless, the combustion of torrefied wood pellets is considered to be carbon-neutral.

The BECCS scenario (the third stacked bar on the left) demonstrates that using bioenergy as fuel and capturing emissions with a CCS plant can effectively decarbonize an iron and steel plant. This intervention reaches near net-zero emissions, and therefore less carbon removal by DACCS was needed. This confirms the study by Tanzer et al.,^10^ who also show that high bioenergy use and high capture rates in a BF/BOF steel plant will result in carbon-positive steelmaking at 0.1 tonne CO_2_ per tonne of HRC.

When the two DRI/EAF case studies are compared (represented by the last two stacked bars on the right), the key difference lies in the electricity consumption of each process. Specifically, the EAF process alone consumes 800 kWh per tonne of crude steel. For DRI-NG, the electricity consumption is 135.5 kWh per tonne of sponge iron, while for DRI-H_2_, it is significantly higher at 3500 kWh per tonne of sponge iron. The high energy demand for hydrogen production is what makes DRI-NG a competitive option when hydrogen is not sourced from renewable energy. This study assumes that the electricity is sourced from the UK grid, which, according to EcoInvent 3.9.1, has one of the lowest carbon intensities ever recorded at 293.28 g of CO_2eq_ per kWh.

#### Future Scenarios

SSPs describe potential future socioeconomic and technologic developments. Meanwhile, RCPs target radiative forcing levels by the year 2100 relative to preindustrial values, measured in watts per square meter. They include projections for emissions and concentrations of GHGs and aerosols as well as land use/land cover. SSPs complement RCPs by providing narratives about how global society, demographics, and economies may evolve over the next century and how these factors influence emissions and climate impacts. The SSP2-RCP2.6 scenario envisions a world where there is a chance of limiting global warming to 2 °C above preindustrial levels by 2050, while the SSP2-RCP1.9 scenario provides a 66% chance of limiting global warming to 1.5 °C above preindustrial levels. Under both future scenarios, emissions in all case studies drop significantly compared with the conventional LCA results. Notably, the BF/BOF (BECCS) case study achieves net negative emissions on its own, eliminating the need for additional DACCS (or other carbon removal).

The key difference to note is that the overall CO_2eq_ emissions of the baseline cases, after incorporation of DACCS into each system, are lower than those reported in conventional LCA results. This reduction is attributed to the higher efficiency of DACCS in future scenarios compared to current time scenarios, which stems from the assumptions of increased electrification and decarbonization in those prospective scenarios.

**Figure 6:**
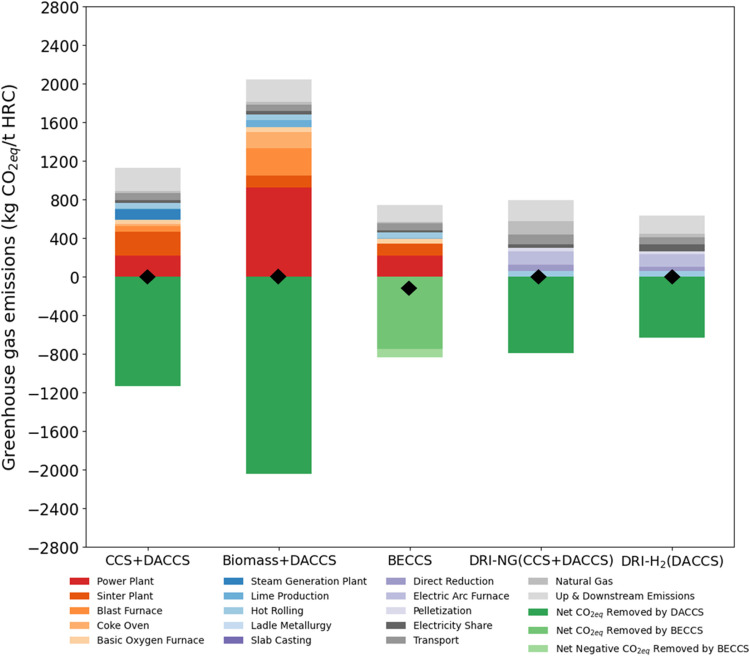
SSP2-RCP2.6 (2050) GHG emissions (kg of CO_2eq_/tonne of HRC) for the net-zero case studies broken down into the origin of the emissions. Note that the BF/BOF (BECCS) case study reaches net negative. The emissions of DRI-H_2_ drop to lower than DRI-NG emissions. (⧫) shows the net-zero CO_2eq_ emissions point.

**Figure 7:**
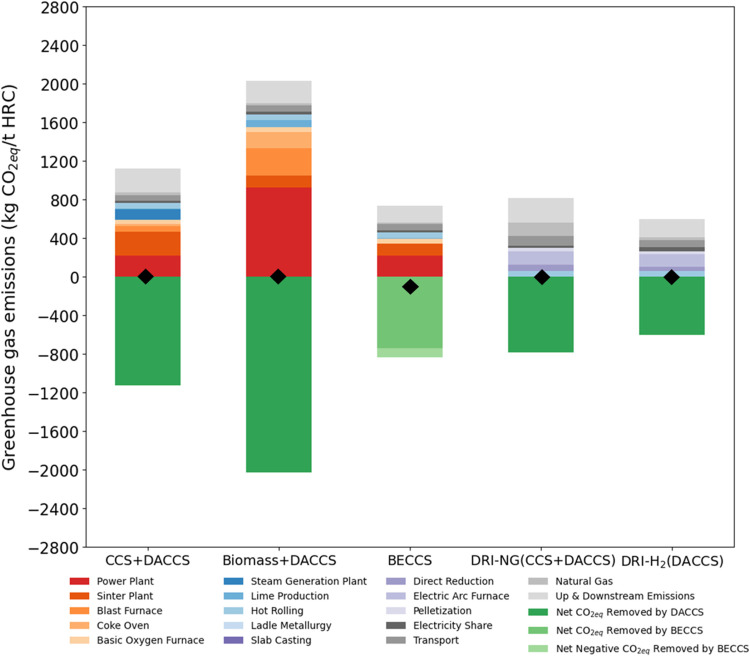
SSP2-RCP1.9 (2050). GHG emissions (kg CO_2eq_/tonne HRC) for the net-zero case studies broken down into the origin of the emissions. Note that the BF/BOF (BECCS) case study reaches the net negative. The emissions of DRI-H_2_ drop to lower than DRI-NG emissions. (⧫) shows the net-zero CO_2eq_ emissions point.

The results of the SSP2-RCP1.9 scenario show slightly lower emissions compared to the SSP2-RCP2.6 scenario. The difference stems from the fact that both scenarios follow similar pathways to reach net-zero, but SSP2-RCP1.9 implements more stringent measures and targets to achieve even lower emissions. The hotspot analysis for both future scenarios is presented in Figures 6 and 7.

#### Non-Climate Change Environmental Trade-Offs

**Figure 8:**
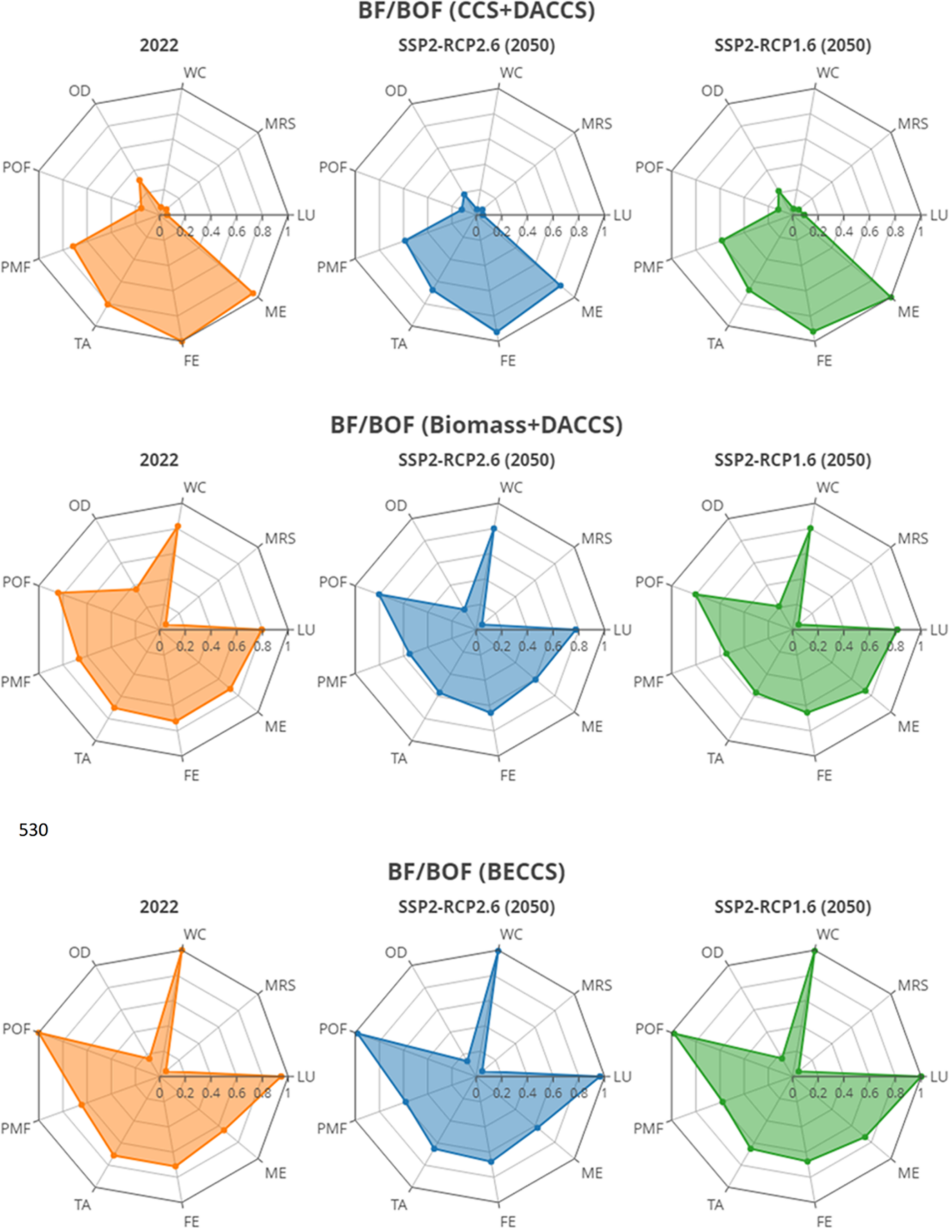

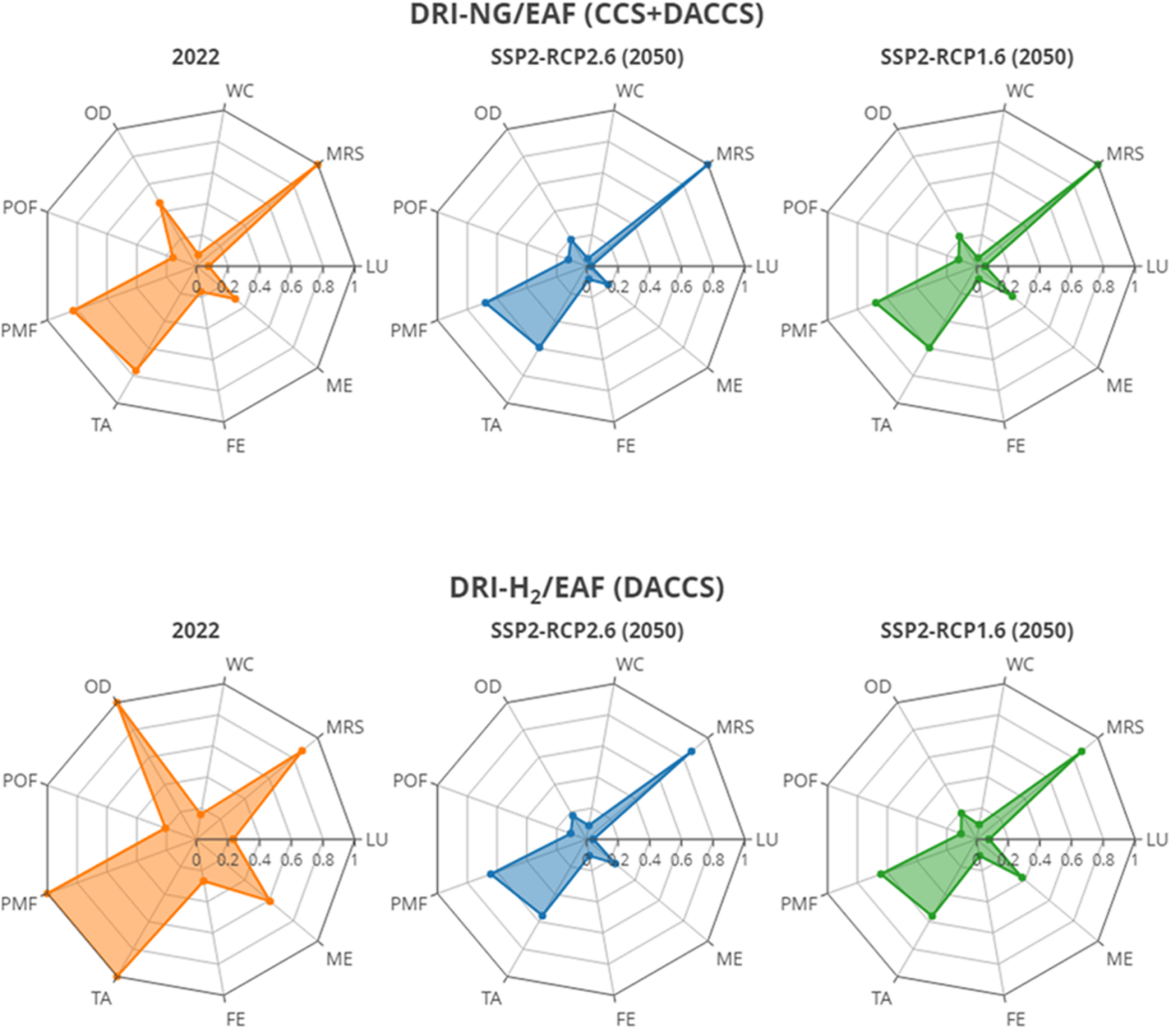
Radar graphs illustrating the environmental trade-offs for the net-zero case studies on nine selected environmental impacts. WC (Water Consumption), MRS (Mineral Resources Scarcity), LU (Land Use), ME (Marine Eutrophication), FE (Freshwater Eutrophication), TA (Terrestrial Acidification), PMF (Particulate Matter Formation), POF (Photochemical Oxidant Formation), and OD (Ozone Depletion).

The key methodological benefit of the net-zero LCA framework is that it allows studying non-climate change environmental impact categories for like-for-like systems (here, net-zero-CO_2eq_ steelmaking) to help identify the most sustainable pathways to net-zero. Figure 8 shows results against the nine selected environmental impact categories (normalized to 1 using the highest environmental impact across all case studies and scenarios). A greater colored area demonstrates an overall higher environmental burden. Each row in Figure 8 shows how each one of the net-zero configurations performs across the different environmental impact categories by changing the background scenarios.

Generally, it is observed that the lowest environmental burdens can be achieved by the DRI/EAF configurations, while they do show substantial impacts on mineral resources scarcity and in 2022 also on terrestrial acidification and particulate matter formation. Both DRI/EAF options show the highest scores across case studies on mineral resources scarcity because DRI/EAF relies heavily on iron ore concentrate used to produce iron ore pellets, while BF/BOF chiefly relies on iron ore and hard coal. Particulate matter formation and terrestrial acidification are high for the 2022 DRI/EAF cases- stemming chiefly from emission of NO_*x*_, NH_3_, and SO_2_ or primary PM_2.5_ to the atmosphere and the deposition of nutrients like nitrogen and sulfur in soil, respectively. Natural gas-fueled DRI with CCS and DACCS shows very similar environmental performance to hydrogen-fueled DRI with DACCS, suggesting investing in either could be a sensible pathway to net-zero. The last row of the figure demonstrates that, for 2022 technology and electricity grid, hydrogen-based DRI may not be the most sustainable option, while in the 2050 scenarios, it will be.

The BF/BOF (CCS + DACCS) case study has the highest impact on freshwater and marine eutrophication in each scenario, primarily due to spoils from hard coal mining and, to a lesser extent, the treatment of wastewater and BF slag. In contrast, the other two BF/BOF case studies have mitigated this impact by substituting part of the hard coal with bioenergy. Additionally, the emissions from storing the CO_2_ (drilling a deep borehole and constructing pipelines) and constructing and demolition of a DACCS plant also contribute to eutrophication.

The BF/BOF (Biomass + DACCS) and (BECCS) case studies expectedly perform worst from the perspective of land use, as the land transformation and use from forest plantations was considered in the system boundary. These two configurations also show that biomass substitution led to higher values in photochemical ozone formation because the biomass torrefaction process releases nonmethane volatile organic compounds (NMVOC) which strongly impact ground-level ozone formation.

The BF/BOF (BECCS) case study is consuming the most water, among others for pretreatment of biomass like removing impurities prior to going through torrefaction (7.92 kg of water per 1 kg of biomass), whereas production of 1 kg of hydrogen using an electrochemical cell needs 0.63 kg of water.

Summarizing, and as clearly shown in Figures S12–S14, DRI/EAF appears to be the route to net-zero with the lowest ecosystem burdens, while BF/BOF fuel substitution with bioenergy plus CCS appears to have the highest impact on the ecosystem end point indicator. Figures S12–S14 also suggest that all case studies score equal on human health, while the DRI/EAF cases have the largest burden on natural resources. On a final note, the environmental impacts are similar for NG-based DRI/EAF and H_2_-based DRI/EAF, suggesting that in regions where natural gas is readily available, the most effective strategy for decarbonizing the iron and steelmaking industry may be to implement DRI-NG/EAF plants in combination with CCS and DACCS.

### Discussion

In this study, we have undertaken LCA of the same case studies for current time and future scenarios by implementing the net-zero-CO_2eq_-emissions framework. The advantage of this method was that it allowed us to restrain the GHG emissions to net-zero and then compare the systems through other non-climate change impact categories. For doing this, we needed a complete inventory of emissions other than the carbon dioxide to be able to run such holistic assessment.

A key assumption in this study is that the foreground database for all case studies remains static across both current and future scenarios, with the only change being the background database, which shifts from EcoInvent version 3.9.1 to Premise. A study by Weckenborg et al.^25^ explored pathways for transforming Germany’s steel industry toward low-carbon production using a prospective assessment method by modifying foreground data via modeling transformation trends for each production route with a linear programming model. This model evaluated the life cycle inventory levels for each year, considering material and energy flows between the routes. The model aimed to minimize the annual variable costs of crude steel production with predefined annual capital and operational costs. Additionally, they adjusted the background data using three environmental scenarios based on the REMIND model within the Premise. Their findings align with the results presented here. They indicate that transitioning from BF/BOF to DRI-NG/EAF could reduce GHG emissions from 2.1 to 0.88 tons of CO_2eq_ per ton of crude steel between 2022 and 2050. Furthermore, they project that by 2050, the GHG intensity of crude steel production via DRI-H_2_/EAF could reach as low as 0.42 tons of CO_2eq_ per ton of crude steel. They, however, highlight that natural gas-based DRI could be a favorable option compared to H_2_-DRI primarily due to its economic advantages. Using hydrogen in the DRI plant starting specifically in 2025 would increase the average crude steel production cost by 10% compared to natural gas, whereas natural gas is readily available in the near term, supporting the economic viability of DRI-NG during the initial stages of transformation to green steel industry.

In this study, we did not examine the costs and economics of steel decarbonization pathways. However, for context, a recent study by Lopez et al.^26^ examined the techno-economic options for three European countries—Germany, Spain, and Finland—and five different steel supply chain configurations compared to local production. The study concluded that by 2030, green hydrogen-based steelmaking via the DRI-H_2_/EAF route could be economically competitive with conventional BF/BOF steelmaking, particularly with the implementation of GHG emission pricing mechanisms. Vogl et al.^27^ also indicated that DRI-H_2_/EAF could compete with BF/BOF steelmaking at carbon prices of 34–68 €/t_CO_2__ and electricity costs of 40 €/MWhel (while we note this may be unrealistically low for the European context). However, without these pricing mechanisms, the European steel industry may be reluctant to transition to green steelmaking, despite recent investments signaling interest in green alternatives. For instance, in Finland, BF/BOF steelmaking could remain the least costly option until 2050 if full domestic supply chains are retained. Transitioning to green steelmaking would require substantial investments not only in new steelmaking capacity but also in the power sector. Projections indicate significant hydrogen and electricity demands for a full transition to green steelmaking in Europe. Specifically, the region of Europe would need 207, 366, and 421 TWh of hydrogen in 2030, 2040, and 2050, respectively.^28^ Additionally, for northern European countries, a transition to green steel would necessitate increases in electricity generation of well over 200 TWh.^29^

### Conclusion and Future Work

This study conducted a comprehensive attributional LCA on the full value chain of net-zero iron and steelmaking in the United Kingdom by applying the net-zero CO_2eq_ emissions framework. This study aimed to fill a critical gap by finding the most environmentally benign pathway to reach net-zero CO_2eq_ steelmaking by 2050 and to assess potential environmental trade-offs. Five carbon emission mitigating strategies were designed and implemented in selected steelmaking routes to evaluate their effectiveness in achieving net-zero GHG emissions. The environmental performance of each decarbonization option was first examined using a conventional LCA for the “current time” (2022) after which a prospective LCA based on IAM scenarios and using the Premise tool was undertaken to project case study performance to 2050.

The main findings indicate that in the “current time” for both non-net-zero and net-zero case studies, the natural gas-fired direct reduced iron with electric arc furnace configuration is the most favorable option. This is because the electricity needed for the production of hydrogen with the current UK grid mix applied to the hydrogen-based direct reduced iron with electric arc furnace route shifts environmental burdens upward. However, in the 2050 scenarios, the overall GHG emissions of all case studies dropped significantly compared to those in the 2022 scenario. Notably, the case study on bioenergy with CCS integrated in a blast furnace with a basic oxygen route not only achieved net-zero GHG emissions but also became slightly net negative. The emissions from the hydrogen-based direct reduced iron with electric arc furnace decreased to around 599 kg CO_2eq_ per tonne of HRC, which is lower than those of natural gas-fired direct reduced iron with CCS (about 747 kg CO_2eq_ per tonne of HRC) and without CCS (around 1347 kg CO_2eq_ per tonne of HRC). This reduction is attributed to IAM scenarios, which assume significant decarbonization and electrification of technologies and industries, leading to reduced GHG emissions and a significant decrease in the carbon intensity of the electricity grid.

All net-zero-CO_2eq_ steelmaking case studies perform similarly on human health indicators, while the direct reduced iron with electric arc furnace options have relatively 60–82% lower impacts on ecosystem end point indicator than the blast furnace basic oxygen furnace routes. The pathway with the highest impact on the ecosystem end point indicator in net-zero iron and steelmaking is the fuel substitution with bioenergy combined with CCS in the blast furnace with the basic oxygen furnace route. This pathway shows an 80% greater environmental impact compared to other case studies. In the “current time” scenario, the hydrogen-based direct reduced iron with electric arc furnace case study ranks highest on natural resource, indicating that with existing technology and the current electricity grid, natural gas-based direct reduced iron is the most sustainable option.

For industries and countries developing their decarbonization strategies, this means the following (at least, from an environmental point of view). If existing blast furnaces are at end of life and new blast furnaces would have to be constructed, the logical choice is to abandon the blast furnace plus basic oxygen furnace route altogether and opt for direct iron reduction plus electric arc furnaces (to the extent that this can produce the steel qualities desired). This can be natural gas-fired direct reduced iron initially, which may be converted to hydrogen-fired direct reduced iron closer to midcentury. However, if blast furnaces still have many miles on them, it could be more beneficial to implement CCS to reduce emissions and later integrate DACCS or other CDR technologies to offset any remaining hard-to-abate emissions. Decarbonizing West-European steelmaking via imported biomass feeding to blast furnaces appears the environmentally worst solution.

Finally, this paper showed the value of evaluating decarbonization options from a net-zero perspective, as this is the state of play, we are heading toward in one or two investment cycles. The net-zero framework for LCA, especially when combined with prospective LCA, provides a powerful approach to design truly net-zero-CO_2eq_ systems, and evaluate and compare the trade-offs between the other environmental indicators, ensuring we mitigate climate change in the most environmentally benign manner.

## Supporting Information Available

Detailed explanation of the process design (PDF)
Detailed LCI data for each process (XLSX)
