# Supplementary material for: Prospective Life Cycle Assessment Suggests Direct Reduced Iron Is the Most Sustainable Pathway to Net-Zero Steelmaking
Source: Ind Eng Chem Res. 2025 Feb 8;64(7):3871–85. doi: 10.1021/acs.iecr.4c03321 (PMC11843611; doi:10.1021/acs.iecr.4c03321)
Supplement: Supplementary file 1 — ie4c03321_si_001.pdf [file ie4c03321_si_001.pdf]

Supplementary Materials to:

Prospective life cycle assessment suggests direct reduced iron is the most sustainable pathway to net-zero steelmaking

Arezoo Azimi, Mijndert van der Spek*

Research Centre for Carbon Solutions, Heriot Watt University, Edinburgh EH14 4AS, United Kingdom

* Corresponding author's email: mv103@hw.ac.uk

Figure S1 shows the gas flows for the non-net zero BF/BOF (Base case) and BF/BOF (CCS) case studies. It highlights how retrofitting a CCS plant alters the internal gas flows and identifies which gases will be captured.

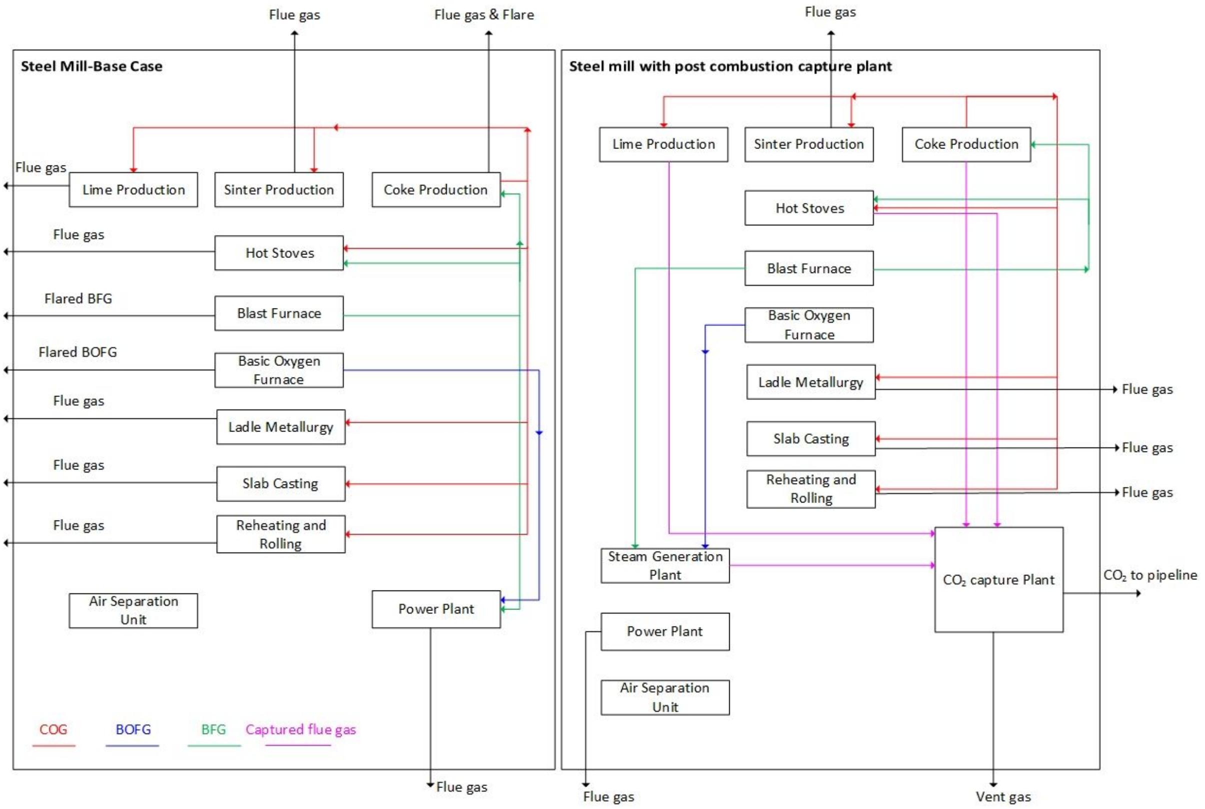

Figure S1. Gas flow in BF/BOF (Base case) and BF/BOF (CCS) case studies. This figure illustrates the internal gas flow through sub processes in an integrated steel mill, both with and without a CCS plant.

Material flow within an integrated BF/BOF steel mill plant with and without a CCS plant is shown in Figure S2.

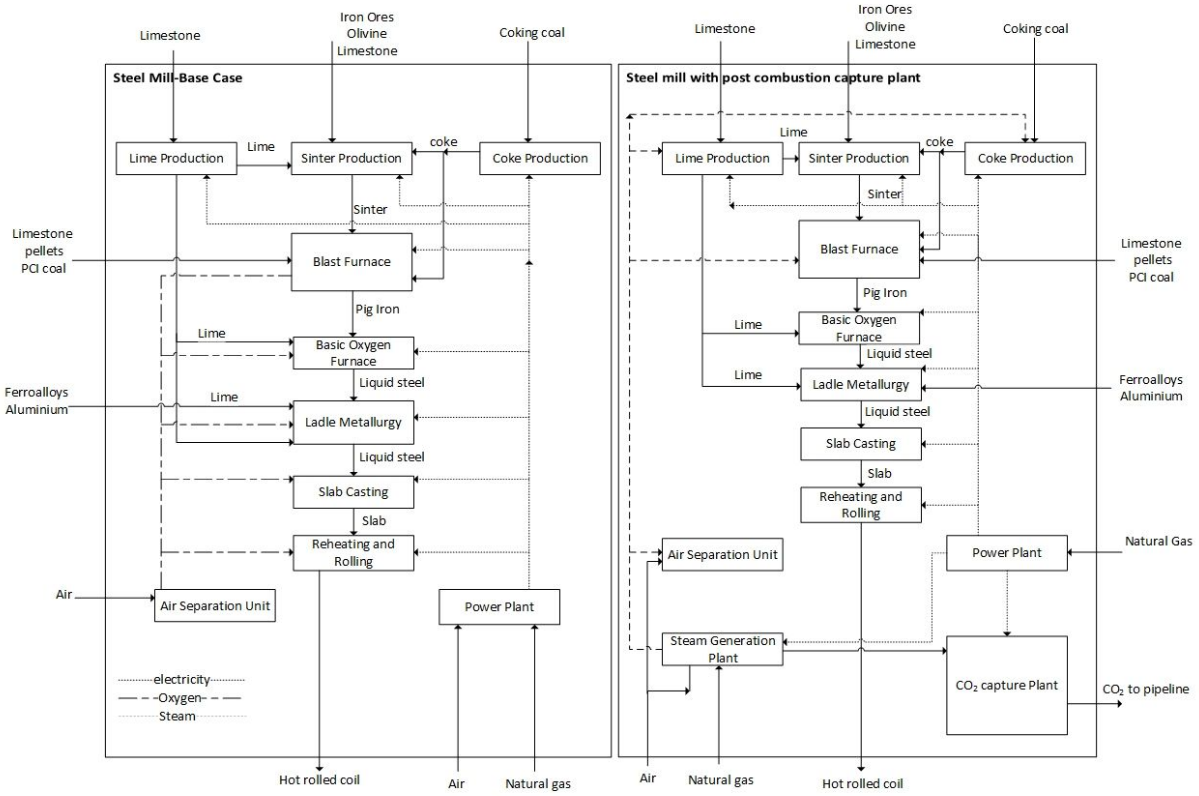

Figure S2. Material flow in BF/BOF (Base case) and BF/BOF (CCS) case studies.

The schematic diagrams of the non-net zero H_2-DRI/EAF and NG-DRI/EAF case studies are presented in Figure S3 and S4.

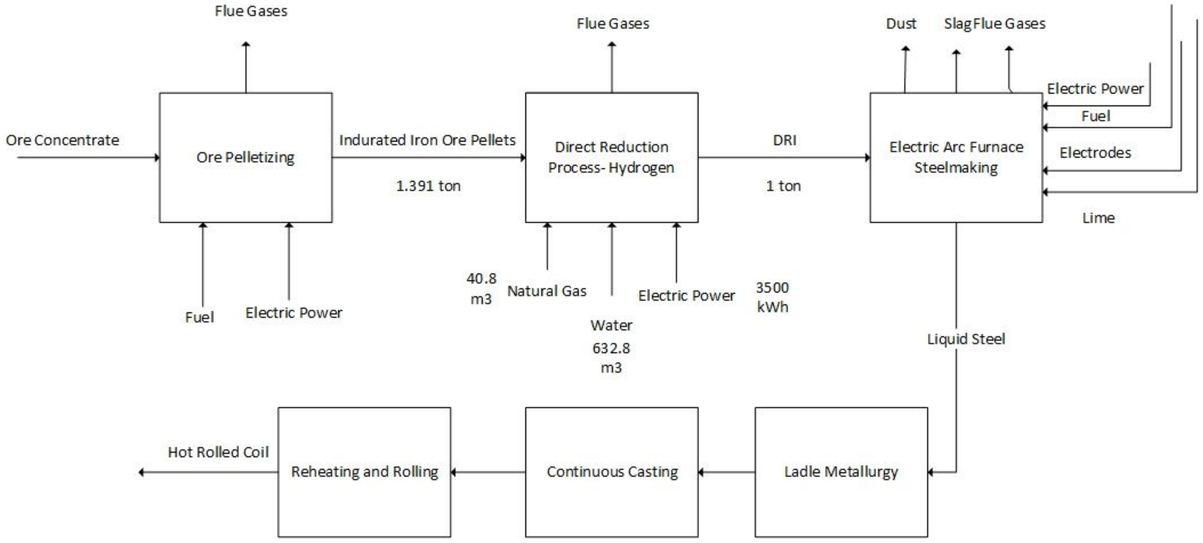

Figure S3. The schematic diagram of DRI-H_2/EAF process. Note the level of electricity and water consumption in the DRI process.

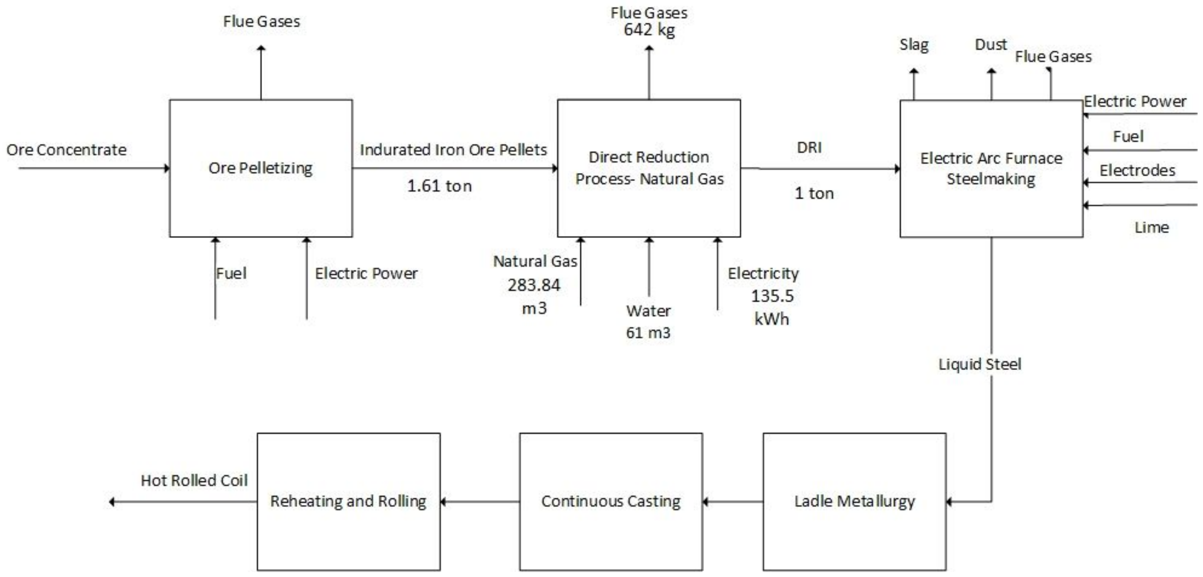

Figure S4. Schematic diagram of the DRI-NG/EAF process. In this case study, water and electricity consumption is lower, however, producing 1 ton of DRI needs higher amounts of iron ore pellets.

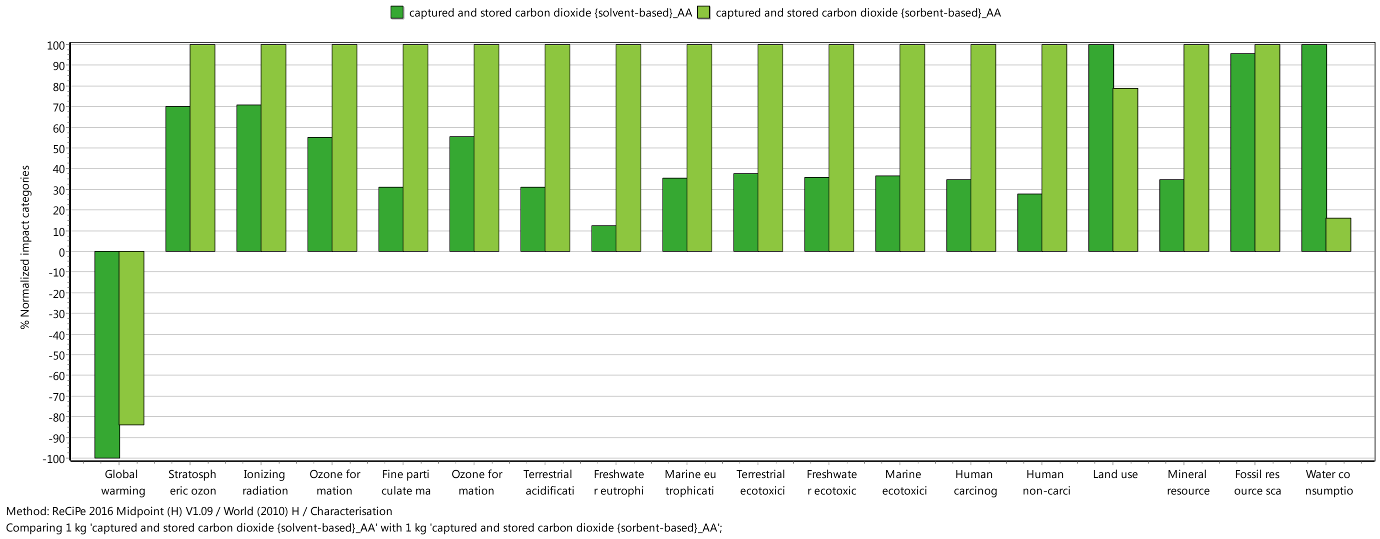

Figure S5. Comparing performance (net removal efficiency) of a sorbent-based DACCS system with solvent-based system when capturing and storing 1 kg of CO_2 across all impact categories.

Non-net zero case studies (hotspot analysis)

The next three figures show the emissions breakdown for all non-net zero cases for current and future scenarios. This analysis helps to understand the emissions of which sub processes have been addressed.

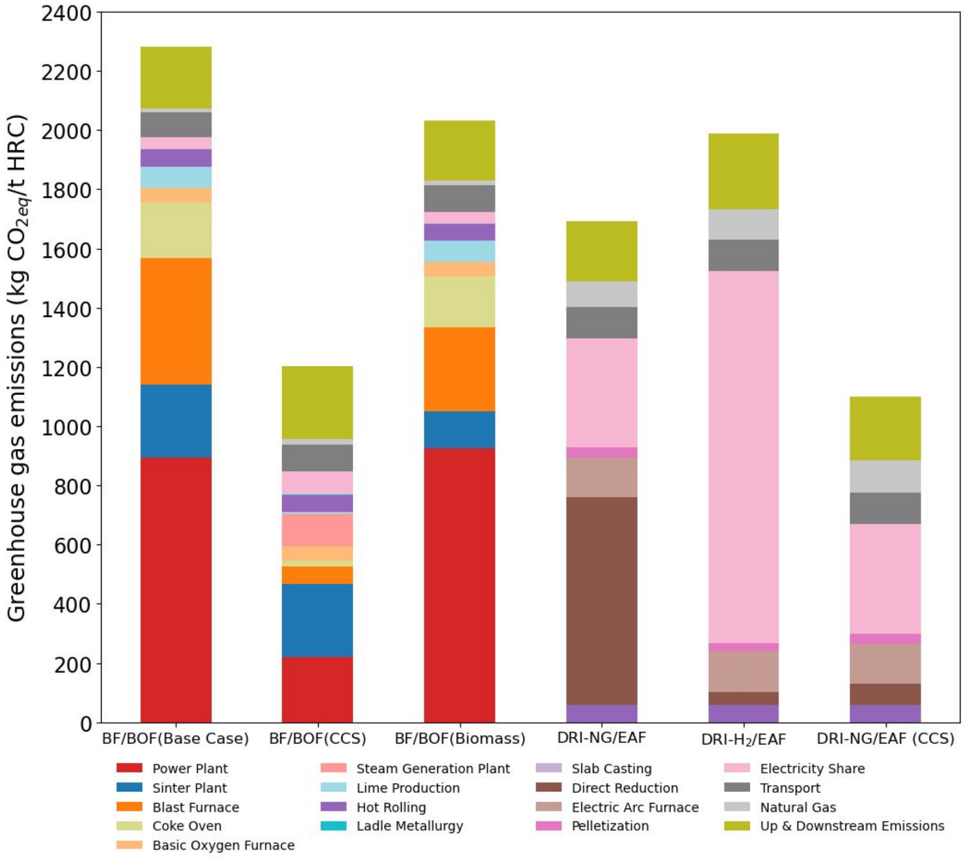

Figure S6. 2022 scenario. GHG emissions (kg CO_{2eq}/tonne HRC) for the non-net zero case studies broken down into the origin of the emissions.

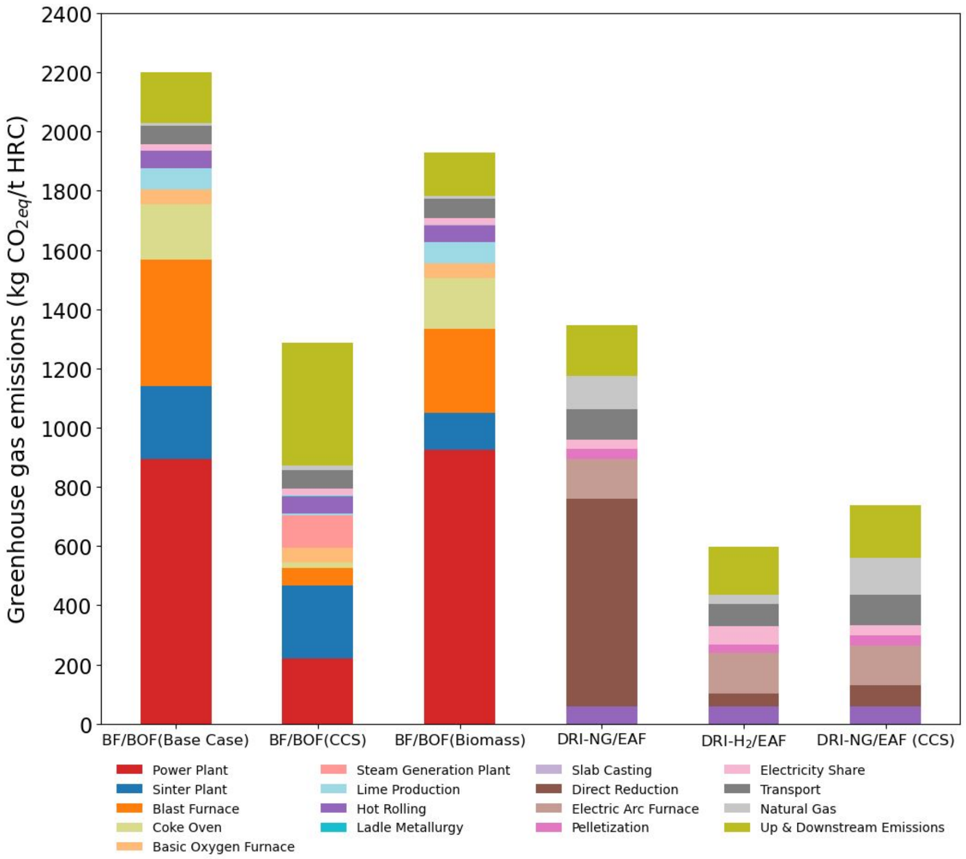

Figure S7. SSP2-RCP2.6 (2050) GHG emissions (kg CO_{2eq}/tonne HRC) for the non-net zero case studies broken down into the origin of the emissions.

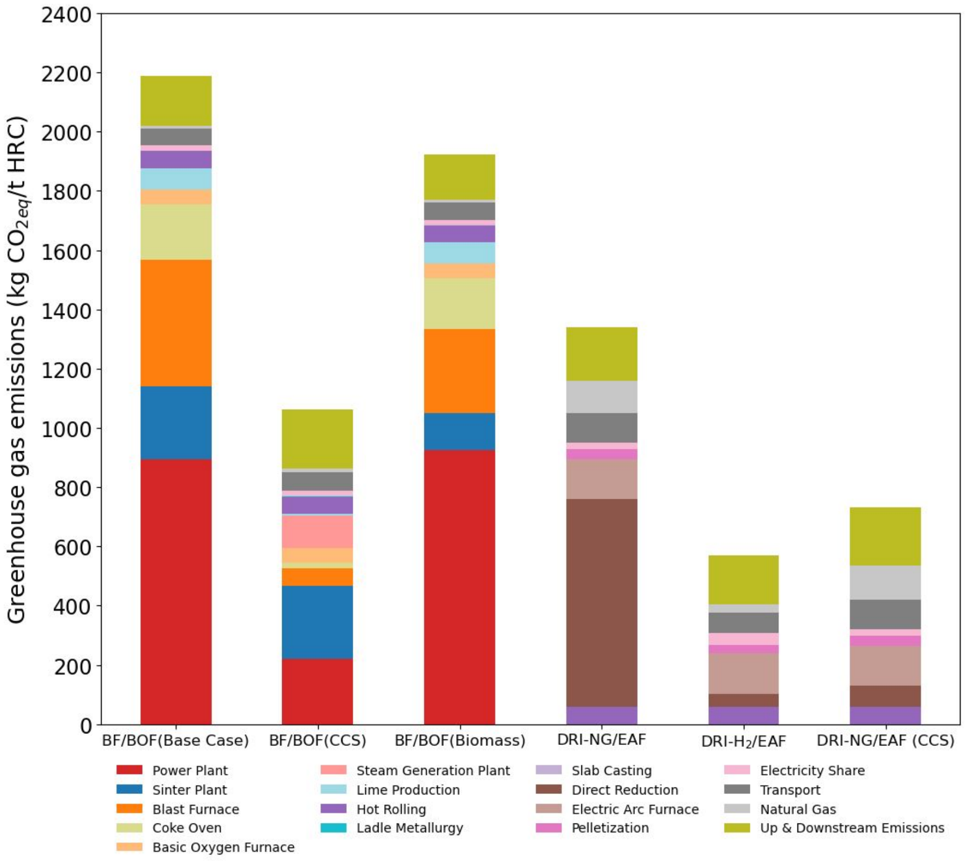

Figure S8. SSP2-RCP1.9 (2050). GHG emissions (kg CO_{2eq}/tonne HRC) for the non-net zero case studies broken down into the origin of the emissions.

Other non-climate change impact categories for non-net zero case studies:

Then, we investigated other impact categories and compared all non-net zero case studies. Figure S9 shows results against the nine selected environmental impact categories, (normalized to 1 using the highest environmental impact across all case studies and scenarios). A greater coloured area demonstrates an overall higher environmental burden. Each row in Figure S9 shows how each one of the non-net zero configurations perform across the different environmental impact categories by changing the background scenarios.

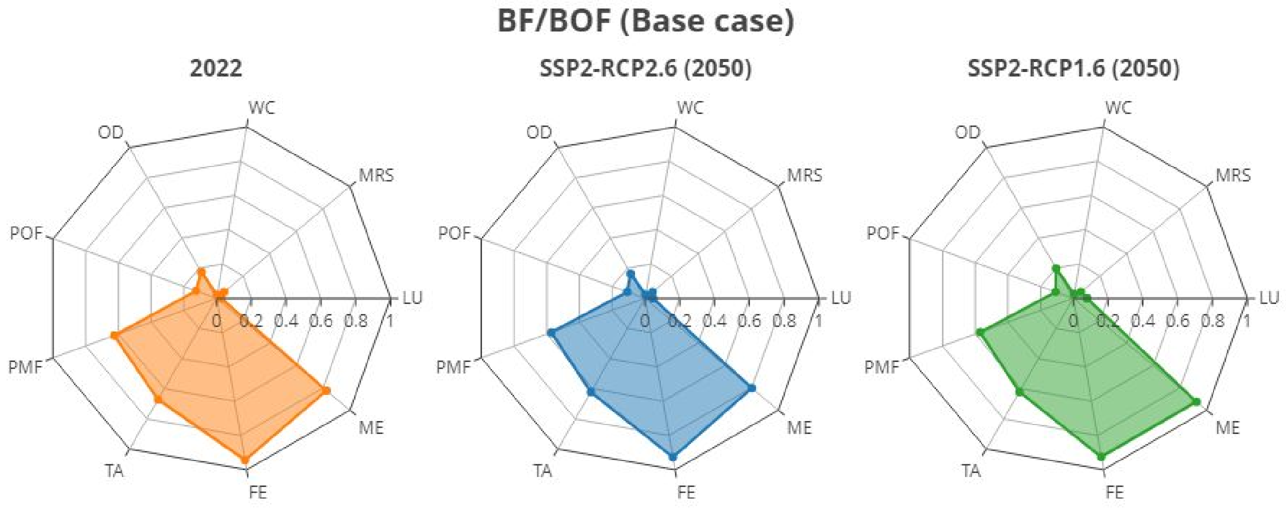
BF/BOF (Base case)
2022
SSP2-RCP2.6 (2050)
SSP2-RCP1.6 (2050)
WC
MRS
LU
ME
FE
TA
PMF
POF
OD
0
0.2
0.4
0.6
0.8
1

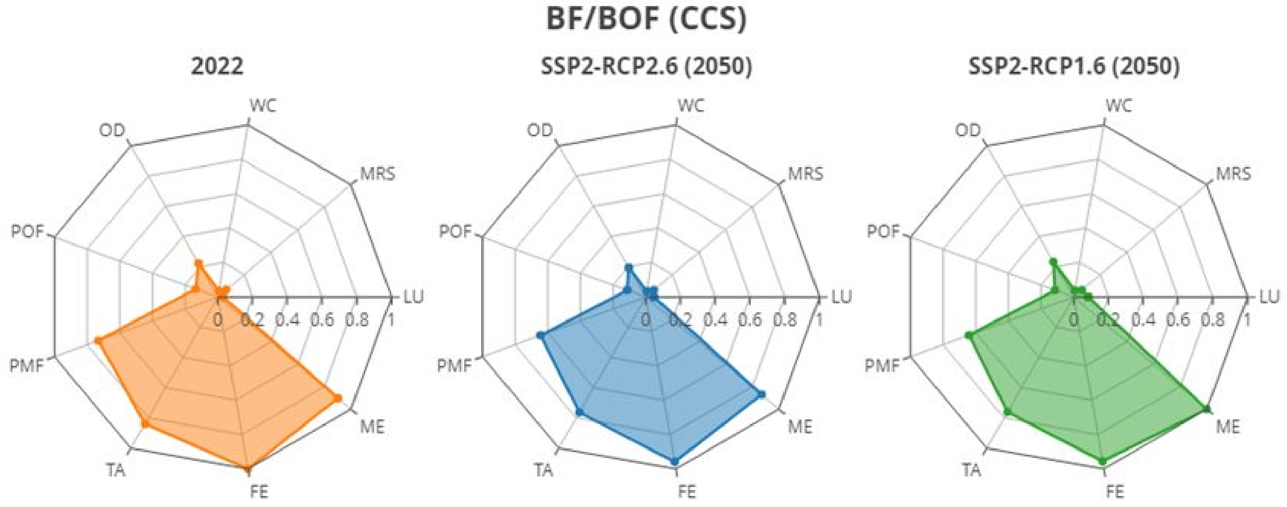
BF/BOF (CCS)
2022
SSP2-RCP2.6 (2050)
SSP2-RCP1.6 (2050)
WC
MRS
LU
ME
FE
TA
PMF
POF
OD
0
0.2
0.4
0.6
0.8
1

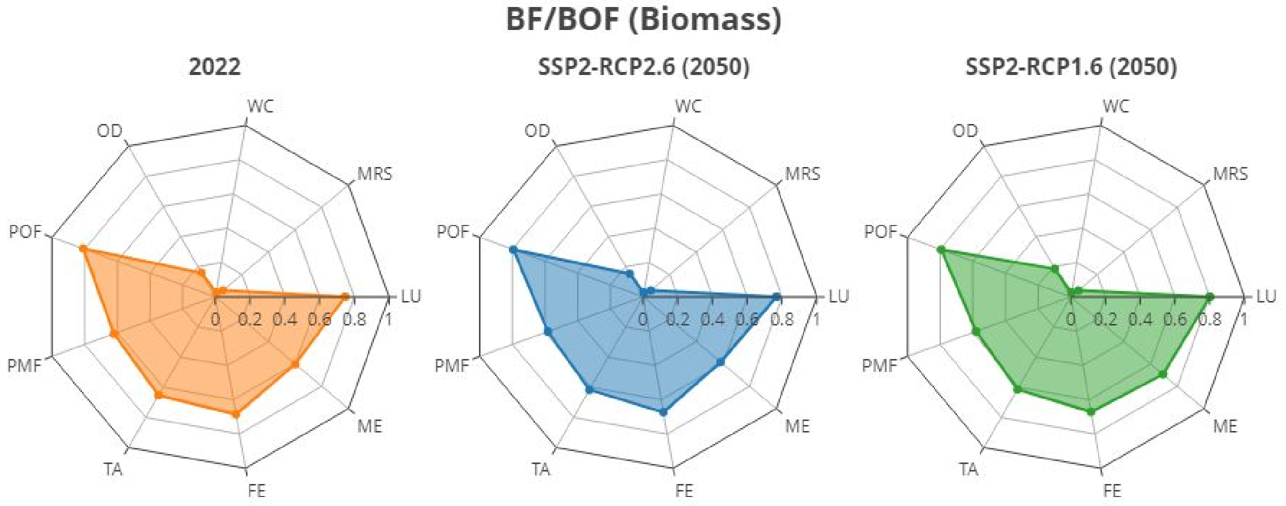
BF/BOF (Biomass)
2022
SSP2-RCP2.6 (2050)
SSP2-RCP1.6 (2050)
WC
MRS
LU
ME
FE
TA
PMF
POF
OD
0
0.2
0.4
0.6
0.8
1

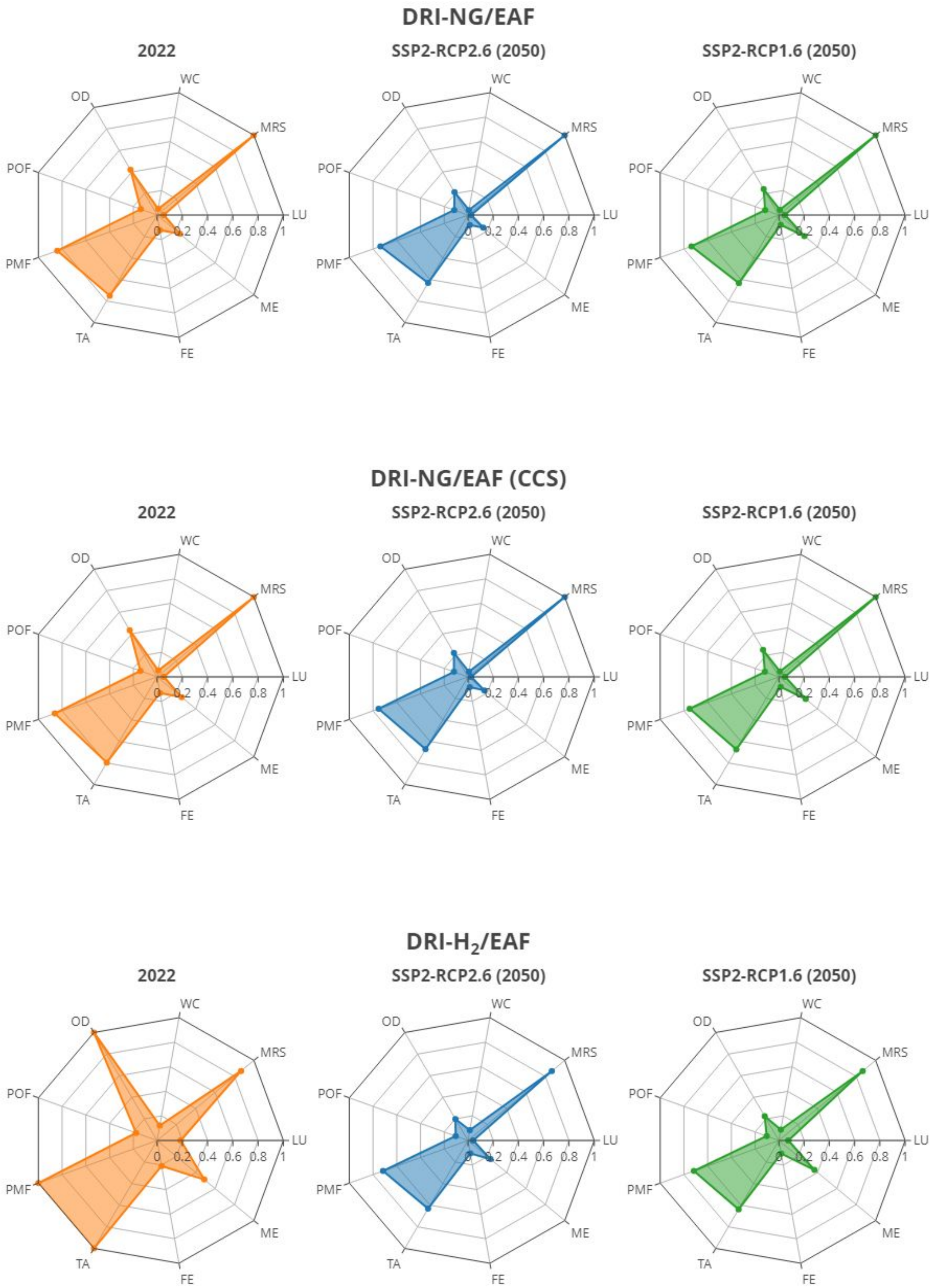

Figure S9. Radar graphs illustrating the environmental trade-offs for the non-net zero case studies on nine selected environmental impacts. WC (Water Consumption), MRS (Mineral Resources Scarcity), LU (Land Use), ME (marine Eutrophication), FE (Freshwater Eutrophication), TA (Terrestrial Acidification), PMF (Particulate Matter Formation), POF (Photochemical Oxidant Formation), and OD (Ozone Depletion).

An additional case study was developed, where the flue gas from power plant is captured by adding a new capture plant on site. The reason that we need an additional capture plant is that the concentration of CO_2 in the other steelmaking gases is quite high, while the CO_2 concentration in the flue gas from power plant is 3.6 vol%. The required electricity for the new capture plant is sourced from UK grid, while the required steam is included in the new capture plant's inventory. The results presented in Figure S10 show that the climate change potential reduces as expected but is still significant.

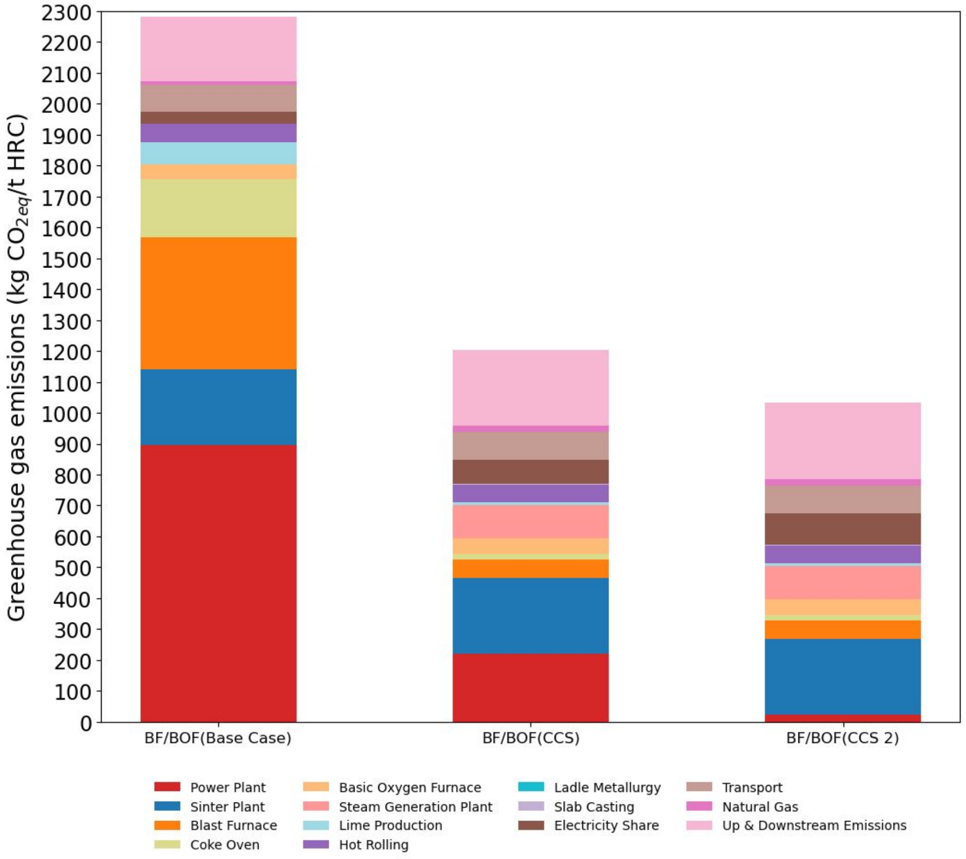

Figure S10. 2022 scenario. GHG emissions (kg CO_{2eq}/tonne HRC) for the non-net zero CCS case studies broken down into the origin of the emissions. BF/BOF (CCS 2) is the case study where 90% of CO_2 emissions from the power plant are captured in addition to emissions from the steelmaking off gases.

Then, we compared this deeper capture case on non-climate change impact categories. It can be observed that adding a new capture plant will reduce the climate change potential but will perform roughly similar on all other impact categories.

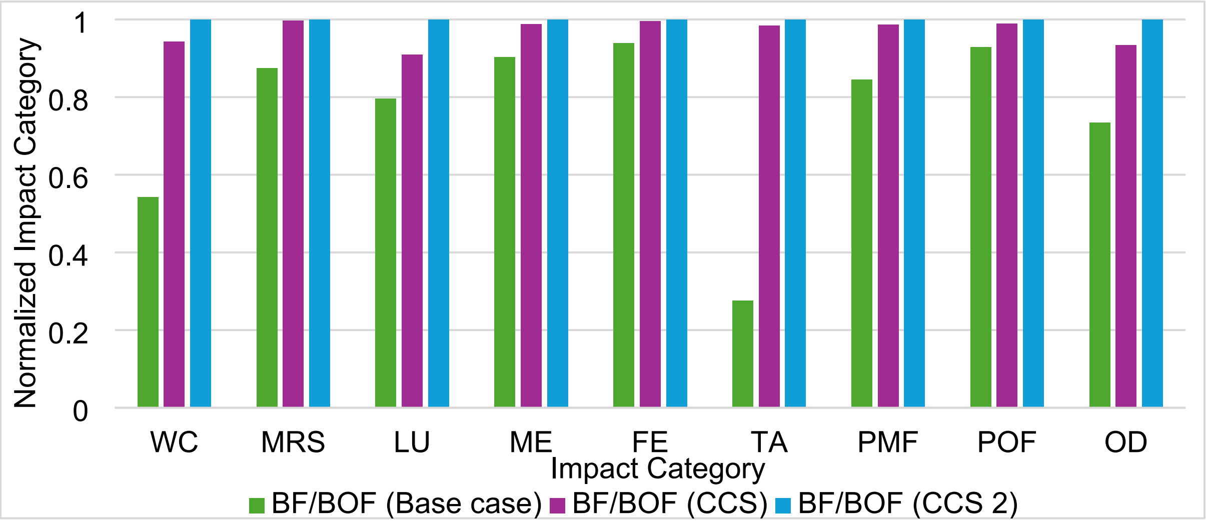

Figure S11. Non-climate change impact categories.

Endpoint indicators for net-zero case studies:

To further assess the systems, we tried to compare all net-zero case studies on endpoint indicators using ReCiPe 2016 v1.03 Endpoint (H) method. The results are shown in Figures S6 to S8. In these figures, the endpoint indicators are normalized to 1 using the highest endpoint indicator across all the net-zero case studies and scenarios.

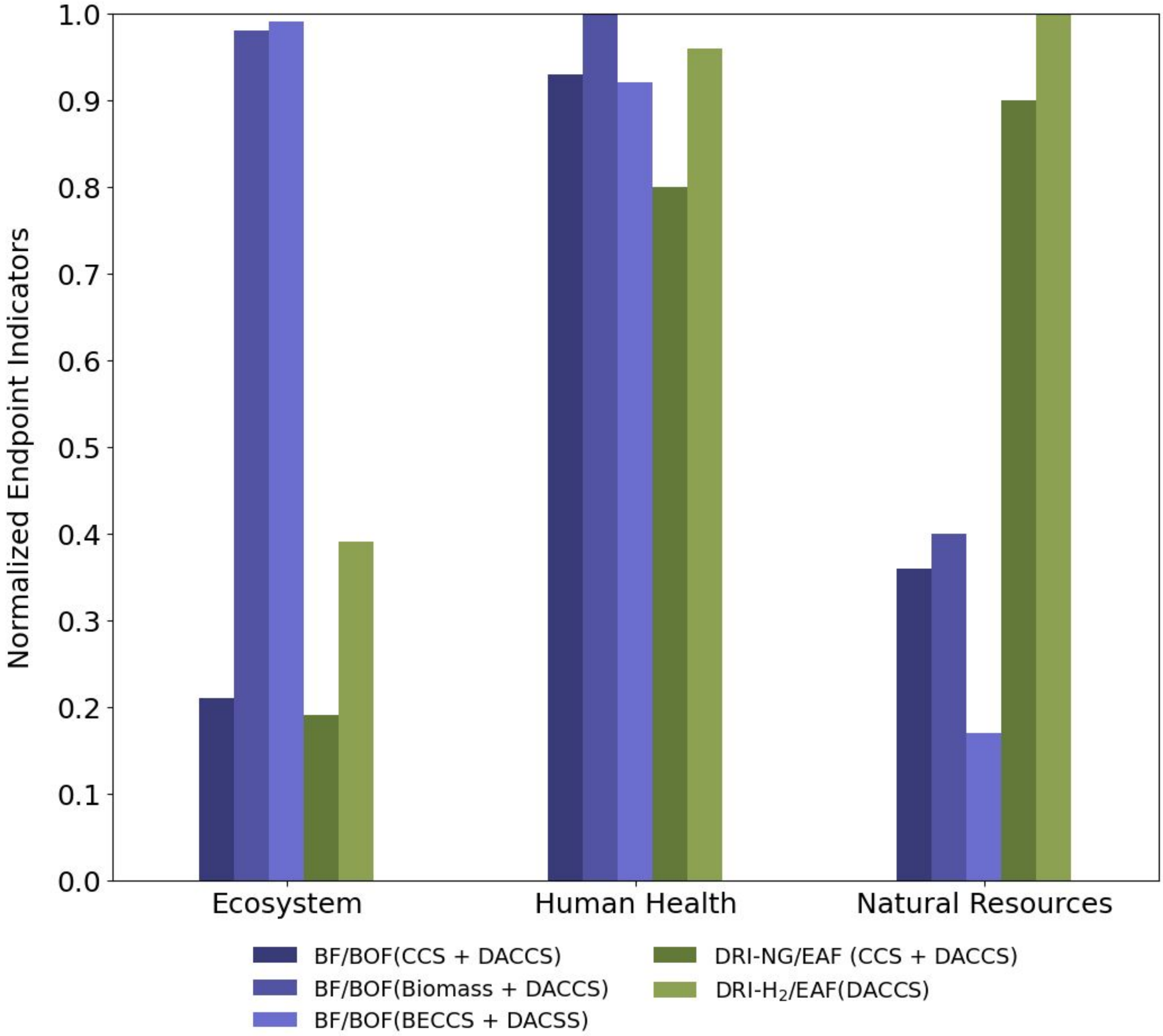

Figure S12. 2022 scenario. Normalized endpoint indicators for net-zero cases. BF/BOF (Biomass+DACCS) has the highest impact on human health and DRI/EAF options use the most natural resources.

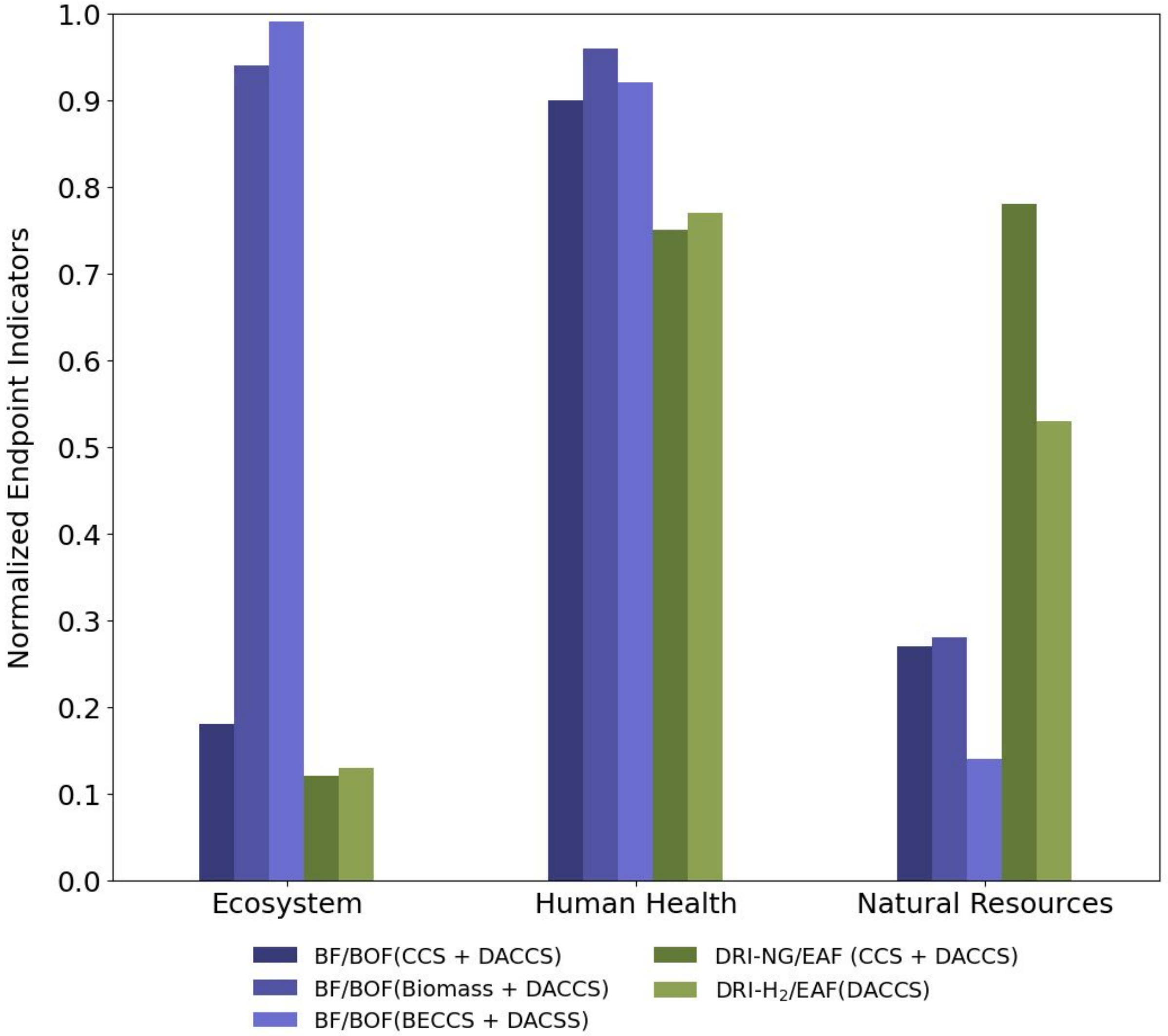

Figure S13. SSP2-RCP2.6 (2050) scenario. Normalized endpoint indicators for net-zero case studies. Over time, the harmful effects of all cases will decrease. The DRI-H_2/EAF impact on natural resources have decreased to lower than that of DRI-NG/EAF in the current time scenario.

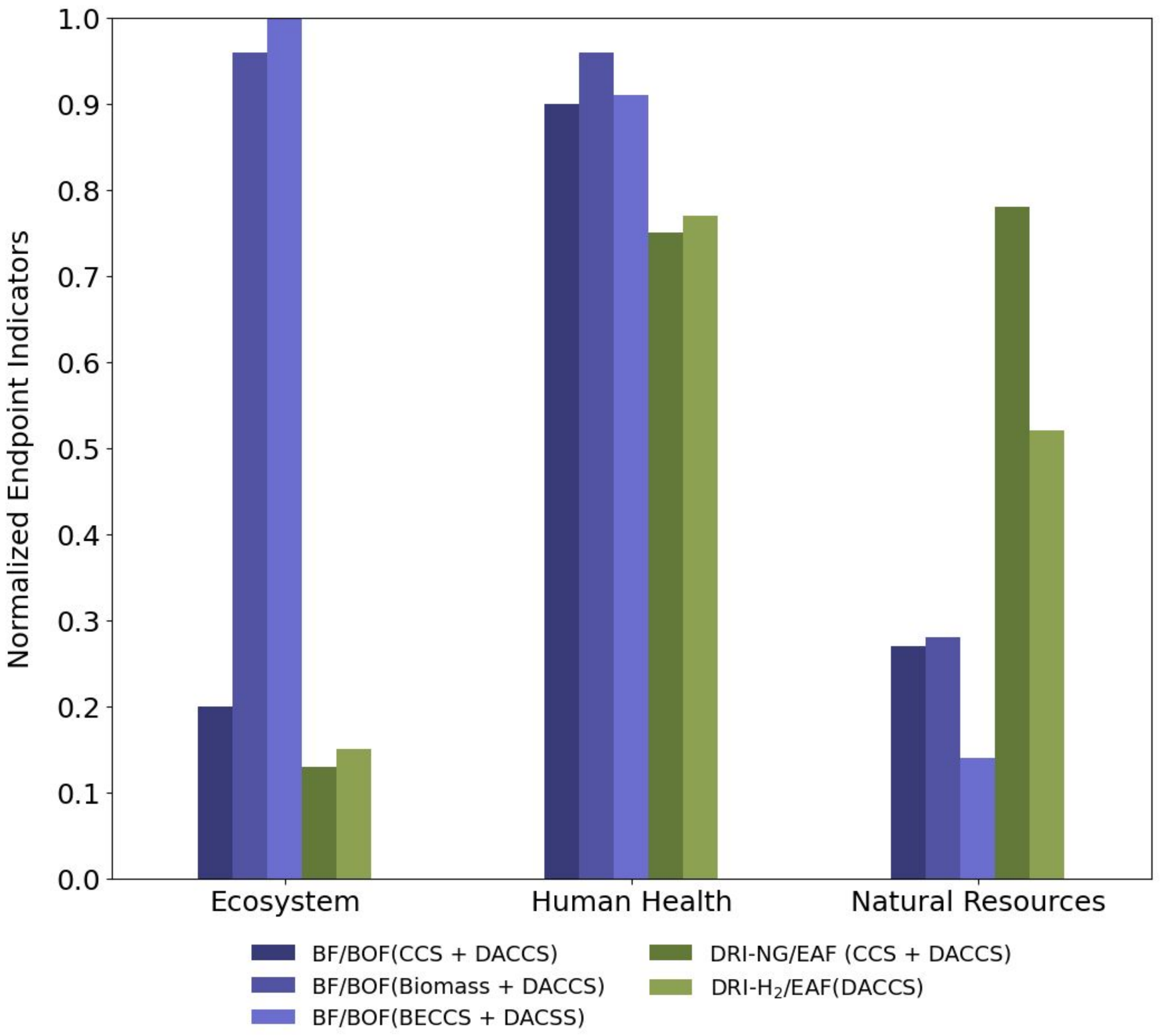

Figure S14. SSP2-RCP1.9 (2050) scenario. Normalized endpoint indicators for net-zero case studies. All case studies are performing almost the same on human health, while the DRI/EAF options have the largest burden on natural resources.

Overall, the study suggests that in the 'current time' scenario, the DRI-NG/EAF is the most sustainable pathway with the lowest impact on the ecosystem. In the future scenarios, overall emissions of all case studies dropped significantly compared to 2022 scenarios. Notably, the case study on bioenergy with CCS integrated in blast furnace with basic oxygen route not only achieved net zero GHG emissions but became slightly net negative. The emissions from the hydrogen-based direct reduced iron with electric arc furnace decreased to levels lower than those of natural-gas fired direct reduced iron, as IAM scenarios assume significant decarbonization and electrification of technologies and industries, leading to reduced GHG emissions and a significant drop in the carbon intensity of the electricity grid.
